# Computational drug repurposing effort for identifying novel hits for the treatment of diseases such as endometriosis, uterine fibroids, and prostate cancer

**DOI:** 10.55730/1300-0527.3667

**Published:** 2024-01-04

**Authors:** Ahmet Buğra ORTAAKARSU, Hilal MEDETALİBEYOĞLU

**Affiliations:** 1 Department of Chemistry, Gazi University, Ankara, Turkiye; 2 Department of Chemistry, Kafkas University, Kars, Turkiye

**Keywords:** Gonadotropin-releasing hormone, prostate cancer, drug repurposing, uterine fibroids, MD simulation, molecular docking analysis

## Abstract

This research aimed to identify potential drug compounds from the ZINC15 molecule database that could effectively treat GnRH1R-related diseases. The study utilized molecular docking and molecular dynamics methods to achieve this goal, which is crucial in drug repurposing research. The virtual screening process involved analyzing known drug compounds using molecular docking. Additionally, molecular dynamics simulations and MM-GBSA were employed to evaluate the stability of the complexes and determine the interactions between the compounds and protein structure. As a result, this study provides significant insights for treating diseases such as endometriosis, uterine fibroids, and prostate cancer related to GnRH1R. The study also involved designing new drugs and identifying necessary molecular scaffolds.

## 1.Introduction

The hypothalamic-pituitary-gonadal triad is a specialized structure on sexual characteristics such as the development of sexual characteristics, reproduction, and preservation of sexual characteristics. The stable operation of this triple structure is of great importance in the reproductive activities of the living organism [1–4]. The most important regulator of this mechanism is the gonadotropin-releasing hormone (GnRH), which is secreted by the hypothalamus and has a peptide structure. GnRH, especially GnRH-1, has a decapeptide structure. The arrangement and structure of amino acid residues is (pyro) Glu-His-Trp-Ser-Tyr-Gly-Leu-Arg-Pro-Gly-NH_2_ [4]. GnRH peptide initiates reproductive functions by activating the receptor and releasing gonadotropin hormones [5]. Follicle-stimulating hormone [6] and luteinizing hormone [7] are essential in reproductive functions, and these hormones are the leading hormones secreted. Changes in GnRH secretion are manifested by physiological and pathological disorders related to reproductive health [1]. There are two isoforms of the GnRH peptide in humans: GnRH-I and GnRH-II. The main effects of these peptides are mediated by the GnRH1 receptor (GnRH1R) [8,9]. GnRH1R begins its initial activation in pituitary gonadotrope cells and is expressed in breast tissue, lymphocytes, prostate cells, and ovaries [10,11]. These expressed structures make GnRH1R a promising therapeutic target in diseases such as uterine fibroids [12], prostate cancer [13], and endometriosis [14].

Many GnRH-analogue agonists have been developed to reduce the activity of receptors in gonadotroph cells to inhibit the secretion of gonadotropin and sex hormones [15]. In addition, GnRH-like antagonists that inhibit GnRH1R signaling have also been synthesized [16–19]. Given the low stability, short half-life, and difficulties in obtaining peptide-based drugs, difficulties in developing small molecule drugs acting on GnRH1R are preferred [10]. The first nonpeptide GnRH1R antagonist was reported by Abbot Laboratories [20]. Other small-molecule medicines have been reported in ongoing studies. The best known of these is elagolix, a uracil derivative. U.S. Food and Drug Administration approves elagolix administration for treating moderate to severe pain caused by endometriosis^1^. The drug is used and developed to treat prostate cancer in men and uterine fibroids in women [21,22]. In addition, sufugolix and relugolix are other drugs developed [23]. The limited number of drugs for treating GnRH1R-related diseases necessitates the repositioning of existing medications.

Drug repositioning, reprofiling, or reassignment is the process of exploring alternative uses for drugs beyond their initially intended purposes that have not been previously authorized [24]. This approach offers an innovative strategy for creating new, cost-effective treatment options developed more quickly [25]. By repurposing existing drugs rather than developing new ones from scratch, the process is less risky, less expensive, and can lead to faster routes to market [26–31]. Consequently, drug repositioning has become a popular approach in drug discovery and development.

This study aimed to predict which 3447 drug compounds in the ZINC15 molecule database [32] could be used for drug repurposing in GnRH1R-related diseases. Molecular docking and molecular dynamics methods are essential in drug repurposing studies [33]. First, virtual screening was performed using the molecular docking method for known drug compounds in the study. Then, molecular dynamics simulations and MM-GBSA were used to examine the complexes’ stability and determine the interactions between the compounds and the protein structure. The study contains new and essential information for treating diseases related to GnRH1R, such as endometriosis, uterine fibroids, and prostate cancer. In addition, the design of new drugs and the identification of critical molecular scaffolds were also performed.

## 2. Computational methods

The study utilized MM-GBSA scoring module within the Maestro software part of the Schrödinger Suite 2023 provided by Schrödinger, Inc., USA. The software package was used to perform various tasks such as protein preparation, preparation of ligands, grid preparation, molecular docking, flexible docking, and MM-GBSA scoring. The procedures were conducted with utmost care and precision in compliance with the best practices in the field. The study findings were based on the reliable and consistent outcomes obtained from the software package.

### 2.1. Selection and preparation of protein structure

Human GnRH1R crystal structure, protein structure with PDB ID:7BR3^2^, was considered suitable due to its resolution of 2.79 Å and the placement of cognate ligand inside the protein structure, specifically elagolix [34] compound. Elagolix is known to be a potent antagonist of cognate ligand GnRH1R, approved by the FDA and used commercially. The appropriate protein structure was imported using Protein Preparation Wizard^3^, included with Maestro [35]. Missing amino acid residues in the imported structure were completed, and their optimized conformations were determined. The “Delete all waters with a distance of 5 Å in ligand” option was checked, and the preprocessing process was started by keeping the other settings as default. Then, minimization was done using the OPLS_2005 force field [36] by optimizing the hydrogen bonds for pH 7.4.

### 2.2. Receptor grid preparation

Incoming cognate ligands were selected from the protein structure to determine the docking region. A grid was created using the default cube size, and amino acid residues that formed hydrogen bond with the protein structure’s ligand were marked. The ligands were instructed to form hydrogen bonds with these marked residues. The scaling factor value was set to 0.5, considering the embedded ligand’s distance from the amino acid residues [37]. The partial charge cut-off value was kept at the default, considering the region’s amino acid residue distance. The grid preparation process was started with all other settings at default.

### 2.3. Preparation of ligands

All compounds used in molecular docking studies were downloaded from the ZINC15 database in SDF format. 1379 FDA-approved compounds and 2068 compounds currently used in the world, although not FDA-approved, were brought into a single file and opened in the Maestro interface. The five most stable conformations of 3447 compounds were selected and prepared for molecular docking at pH 7.4 using the Epik^4^ embedded software in LigPrep^5^. In addition, since the conformation of the ligand that comes with the crystal structure will be used as a template, the protein was removed from the structure, and the same procedures were applied.

### 2.4. Ligand docking

Molecular docking of ligands downloaded from the ZINC15 database was performed by Ligand Docking using Glide^6^. Both rigid and flexible placement algorithms were used to obtain realistic docking scores. In the docking studies, various docking algorithms were utilized in Maestro with flexible and inflexible ligand sampling, including Glide/HTVS, Glide/SP, Glide/XP, and Glide/IFD approaches. In the ligand docking process, the scaling factor was set to 0.5 for the same reasons as in the receiver grid preparation, and the other settings were kept as default. Molecular docking was applied with high-throughput virtual scanning (HTVS), standard sensitivity (SP), and extra sensitivity (XP), respectively. Ligands with the highest docking score were selected each time. The study was carried out with reference and validation of the cognate ligand.

Ligands with the highest docking score in extra sensitivity (XP) were preferred for induced fit docking^7^. This is a more sensitive and advanced analysis method since the docking scores were the highest and MM-GBSA values were the best complexes. Therefore, in the Induced Fit Docking application, all other settings were set as default by selecting the XP option in the redocking tab, and a flexible docking process was started.

### 2.5. Molecular mechanics-generalized born surface area (MM-GBSA)

The MM-GBSA calculation is widely used for evaluating the interaction mechanisms between proteins and ligands, providing insights into their stability [38,39]. The technique employs molecular mechanics (MM) force fields and a generalized Born and surface area continuum (implicit) solvation solvent model to calculate the binding free energy of protein-ligand complexes. This calculation is performed before and after molecular dynamics simulations and is particularly useful for analyzing critical compounds and observing changes in binding energy during the simulation. The MM-GBSA [40] calculation utilized the default setting, which involved rendering all protein atoms rigid while relaxing those of the ligands.

### 2.6. Molecular dynamics simulations

The molecular dynamics method was used to detect conformational changes at the atomic level to observe and analyze how the physical motions of atoms and molecules change [41]. Using the Desmond^8^ module, 100 ns molecular dynamics simulation was applied for eight ligands with the best docking (IFD) scores and MM-GBSA values to determine the stability of complexes formed by molecular docking and to determine the movement of protein structure. In addition, the long-term simulation of the best compound at 1000 ns was performed. This step was essential for modeling the actual effect of the drug compound on GnRH1R.

A membrane system (POPC) was created for molecular dynamics simulations. The membrane was set to contain the alpha-helix structure. The OPLS3e force field was employed to minimize the energy consumption [42]. For dissolution, an orthorhombic box was created, and its dimensions were adjusted to (10 Å × 10 Å × 10 Å) to include the protein structure ultimately. The box was constructed using TIP4P water molecules [43,44]. Ions are added for neutralization. Using the Monte Carlo method, 0.15 M NaCl was added to the constructed orthorhombic box to model the protein structure in its natural environment. The temperature was adjusted with the Nose-Hoover thermostat [45] in the NPT group, around the body temperature of 310 K. The pressure was adjusted to around 1.01 bar with a Martyna-Tobias-Klein (MTK) barostat [46]. Short-range interactions are calculated within 9 Å of the cut-off. Two thousand frames were requested for each simulation. Molecular dynamics simulations were started separately for approximately eighty-two thousand atoms.

## 3. Results and discussion

The evaluation of the GnRH1R structure necessitates a thorough consideration of the binding modes of the elagolix compound, which serves as the protein structure’s cognate ligand. It is crucial to consider the cognate ligand’s position to identify the critical amino acids. The pi-pi interaction of the residue at TYR283 and TYR290 is observed in the cognate ligand’s interaction with the protein structure. Moreover, the pi-cation interaction of the residue at TYR283 provides robust attachment of the uracil core and the molecule’s center to the protein structure. The LYS121 residue also forms hydrogen bond with the carbonyl oxygen in the uracil core, forming the molecule’s center (Figures S1 and S2). When these data are evaluated, it is seen that TYR283, TYR290, and LYS121 residues are the residues in the protein-ligand complex where interaction is required for the antagonistic effect [1]. In addition, studies show that LYS121 and ASP98 residue have an essential role in GnRH1R functioning [47,48]. The region of the ASN305 residue is essential because it is the region of hydrophobic interactions. The proximity of the cognate ligand to the ASN305 region where the trifluoro group is located is important because it is the region where the hydrophobic groups of the new ligands will settle.

The trifluoro group in the cognate ligand is determinative in designing and identifying new ligands as it is a hydrophobic group. The position of the hydrophobic groups in the GnRH1R protein structure is decisive for the new ligands. In addition, the degrees of freedom of the arms attached to the nitrogen atoms in the uracil core are essential for ligand location and pocket placement.

**Figure 1:**
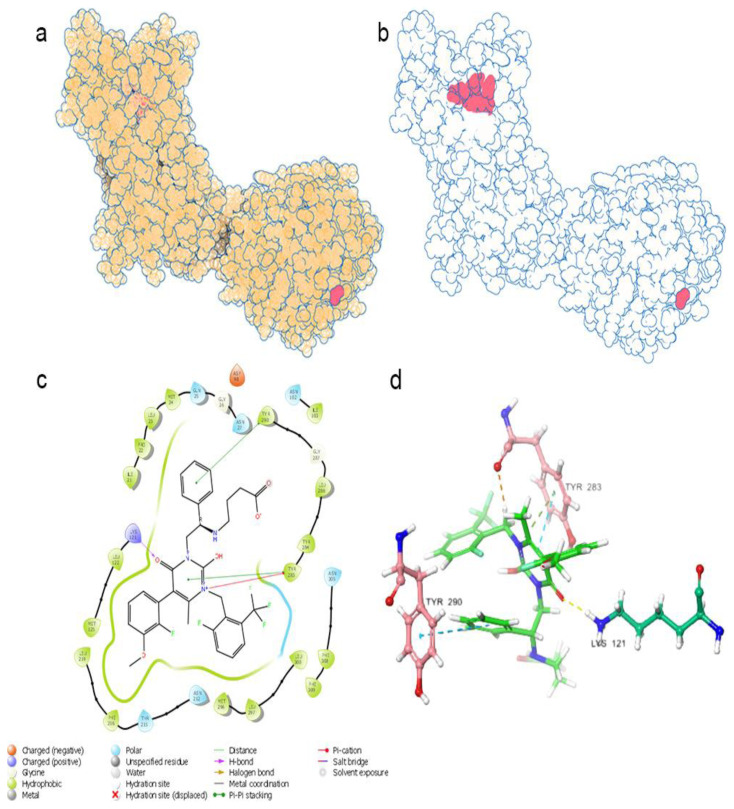
(a) 3D GnRH1R protein structure; (b) 3D position of the cognate ligand (red) in the GnRH1R protein structure - 3D visualization of protein structure was done using Protein Imager [49]; (c) 2D interaction map of the cognate ligand; (d) 3D interaction map of the cognate ligand.

Figure 1 shows the schematic image of the GnRH1R protein structure (Figure 1a). The general view of the location of the elagolix compound, which is a cognate ligand in the GnRH1R structure, in the protein structure is shown schematically in Figure 1b. The two-dimensional interaction map of the elagolix compound and the amino acid residues with which it interacts three-dimensionally are seen in Figures 1c and 1d.

### 3.1. Molecular docking

The study’s findings demonstrate that candesartan and its derivatives interact with critical amino acids and exhibit high binding affinity with the cognate ligand region of the GnRH1R protein structure. Candesartan N2-S-glucuronide performed remarkably well among the derivatives, with a docking score of −16,020 kcal/mol. Moreover, the R form of the same compound achieved a docking score similar to the S form (Table 1). These results indicate that candesartan derivatives hold promise as effective treatment options for conditions that affect the GnRH1R protein structure.

**Figure 2:**
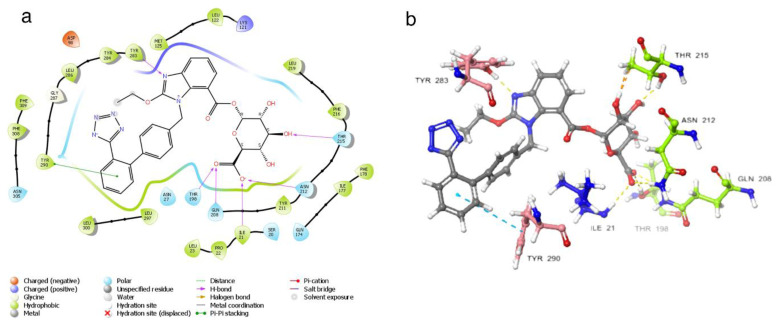
(a) Candesartan O-beta-D-glucuronoside 2D interaction map; (b) candesartan O-beta-D-glucuronoside 3D interaction map.

It is seen that the candesartan O-beta-D-glucuronoside compound strongly interacts with TRY283 and TRY290 residues, which are determined as critical amino acid residues (Figure 2). In addition, THR190, GLN208, ASN212, THR215, and ILE21 have strong interactions with residues. The interaction with residue TYR290 is the pi-pi interaction. The interactions with TRY283, THR190, GLN208, ASN212, THR215, and ILE21 are hydrogen bonds. Instead of the uracil structure in the cognate ligand, the benzimidazole functional group, which has a different binding mode, makes a noncovalent interaction with the TYR283 residue and fixes the center of the molecule to this binding pocket. In addition, strong interactions of the hydrophilic glucuronic acid group with residues TRY283, THR190, GLN208, ASN212, THR215, and ILE21 allow this part of the molecule to be well embedded in the binding pocket. As expected, the ligand with the hydrophobic region near the ASN305 residue interacts hydrophobicly with the LEU300 and LEU297 residues in this region. The aromatic ring groups in the compound are the residues that make hydrophobic interactions in the candesartan O-beta-D-glucuronoside compound (Figure S3).

**Figure 3:**
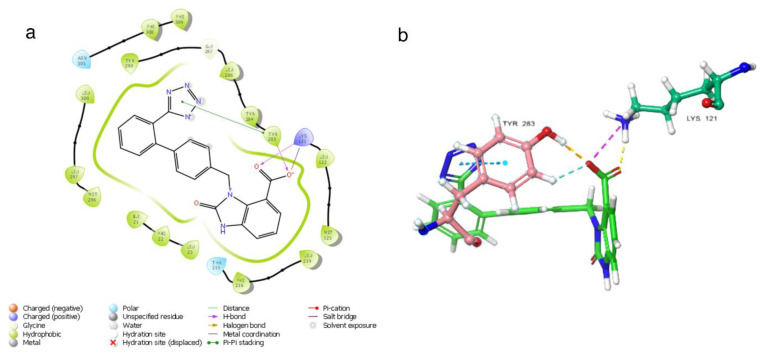
(a) Candesartan 2D interaction map; (b) candesartan 3D interaction map.

In Figure 3, three-dimensional and two-dimensional depictions of the binding of the candesartan to the GnRH1R structure are presented (Figure S4). Noncovalent interactions between the candesartan compound and critical amino acid residues TYR283 and LYS121, also found in the binding modes of the cognate ligand, are observed. The tetrazole ring in the compound interacts with TYR283, one of the critical amino acids, resulting in the fixation of the tetrazole side of the molecule to the protein structure. This mode of interaction differs from that of other compounds. The hydrophobic residues involved in the interaction with the candesartan compound are LEU300 and LEU297, which make hydrophobic contact with the candesartan compound’s aromatic ring. Notably, the conformation of the candesartan compound, when bound to the GnRH1R protein structure, is consistent with its other derivatives.

**Figure 4:**
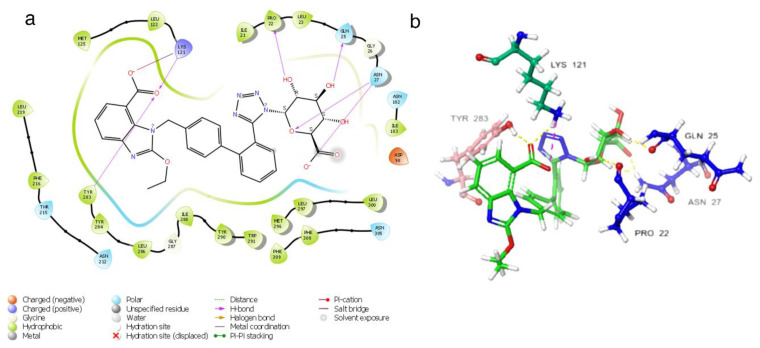
(a) Candesartan N2-S-glucuronide 2D interaction map; (b) candesartan N2-S glucuronide 3D interaction map.

**Figure 5:**
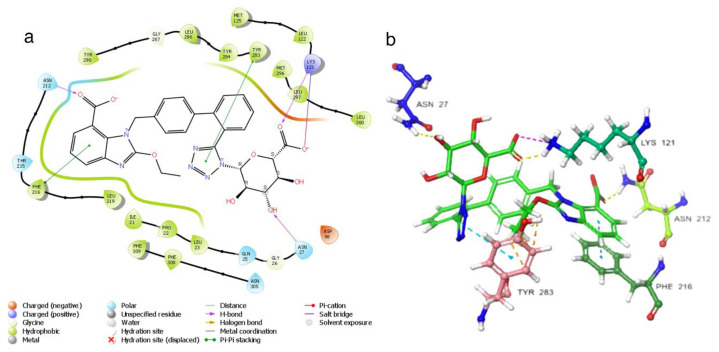
(a) Candesartan N2-R-glucuronide 2D interaction map; (b) candesartan N2-R-glucuronide 3D interaction map.

Candesartan N2-S-glucuronide exhibits strong and multiple interactions with the critical amino acid residues LYS121 and TYR283. These interactions involve the carboxylic acid group linked to benzimidazole, as depicted in Figure 4. Additionally, the candesartan N2-S-glucuronide compound, which achieves the highest docking score (Table 1), interacts with the S-glucuronide group and PRO22, GLN25, and ASN27 residues through hydrogen bonds. The fact that the S-glucuronide group is in the S form shows its importance with much less interaction in Figure 5, where the R form is displayed. It can be seen in Figure 5 that the compound in the R form completely changes the conformation of the ligand in the protein structure.

The candesartan N2-S-glucuronide compound has potential as a potent antagonist due to its ability to cover the channel initiation of the receptor entirely (Figure S5). Additionally, the S-glucuronide group effectively covers the middle region of the protein structure, creating a solid and stable ligand-protein complex. However, the R form of the compound demonstrates a weaker interaction with the protein structure, leading to only partial embedding of the compound in the protein structure.

The candesartan N2-R-glucuronide compound has been observed to form hydrogen bonds with LYS121, ASN212, and ASN27 residues within the protein structure. However, this compound’s number of hydrogen bonds is notably lower than that formed by its S counterpart. In addition (Figure S6), the candesartan N2-R-glucuronide compound exhibits a pi-pi stacking interaction with PHE216 and TYR283 residues and a salt bridge with LYS121. Notably, the binding conformation of the candesartan N2-R-glucuronide compound to the protein structure is distinct from other derivatives of candesartan. Furthermore, unlike other candesartan derivatives, the candesartan N2-R-glucuronide compound does not exhibit hydrophobic interactions with LEU300 and LEU297 residues.

Upon examining the binding modes of the compounds, it is apparent that including the benzimidazole functional group and its associated reactive groups results in favorable interactions at the compound’s binding site. These findings underscore the necessity of utilizing the candesartan N2-S-glucuronide compound in molecular dynamics simulations, which can offer a more comprehensive understanding of its behavior by providing insight into the persistence of its strong interactions over time. In addition, utilizing molecular dynamics simulation techniques is essential in elucidating the stability of these interactions.

### 3.2. Molecular dynamics simulation

For comparison, 100 ns molecular dynamics simulations of the eight compounds with the highest affinity for the GnRH1R protein structure and the elagolix compound were performed. Among these molecular dynamics simulations, the complexes formed by a hit compound and elagolix compound that inhibit the activity of the GnRH1R protein structure were compared with a 1000 ns molecular dynamics simulation.

#### 3.2.1. Trajectory analysis

**Figure 6:**
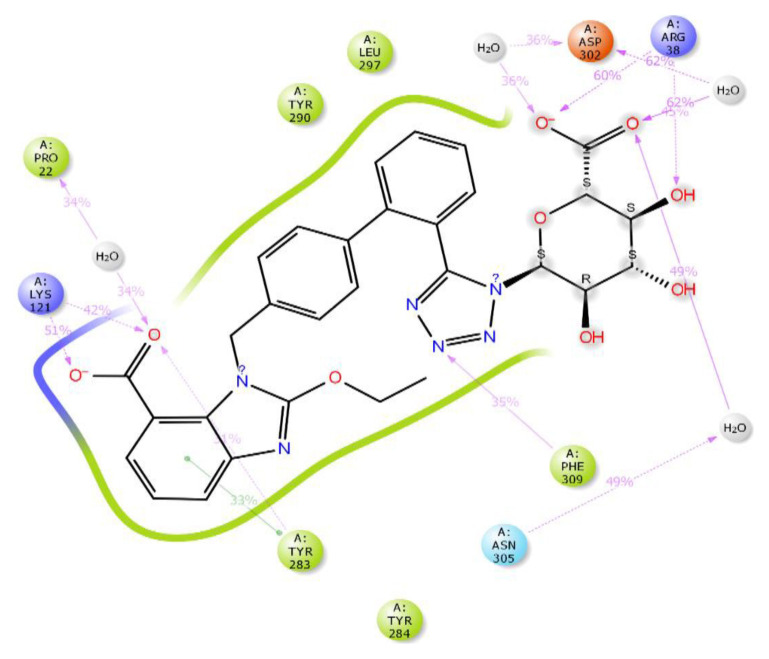
Ligand–residue interactions that persist for more than 30% of M.D. simulation time for 3665.

**Figure 7:**
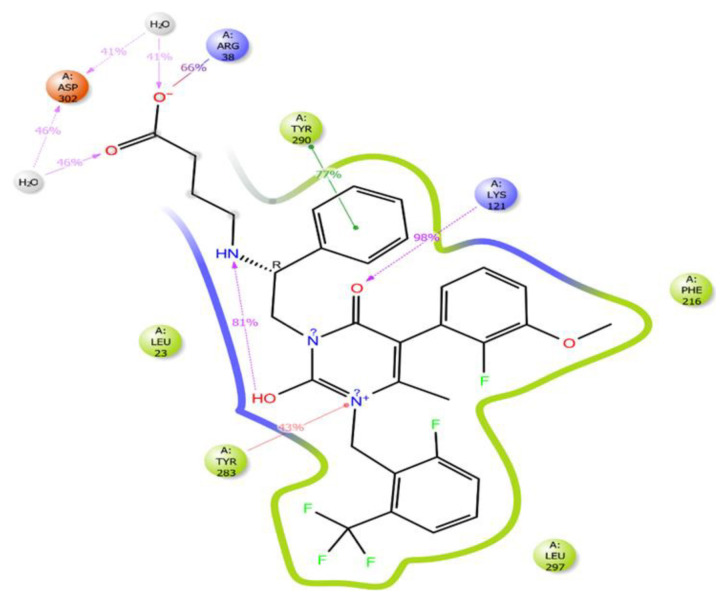
Ligand–residue interactions that persist for more than 30% of M.D. simulation time for elagolix.

The Desmond trajectory clustering tool, with the backbone RMSD matrix as the structural similarity matrix, hierarchical clustering with average linkage for the clustering method, and a merging distance cut-off set at 2.5 Å, was used for this analysis [50]. In addition, the structure with the most significant number of neighbors in the structural family, the centroid structure, was used for representation (Figures 6 and 7).

The study employed molecular dynamics simulations to scrutinize the behavior of four ligands, including elagolix, in a complex with a target protein. Trajectory analysis uncovered that the ligands remained relatively stationary initially, with minimal conformational changes, while the protein underwent more significant changes in selected regions. One of the ligands, coded 2512, exhibited the most conformational changes, particularly in the projection formed by amino acid residues LEU297 and ASN298. Ligand 4054 showed considerable stability throughout the simulation, while ligand 1291 displayed fluctuations in the early stages before attaining stability. The ligand with the best docking scores, coded 3665, interacted with key residues in the protein, including ARG38, which is essential for receptor activity (Table 1). The glucuronide tip of the molecule underwent conformational changes, directing it towards ARG38, while the benzimidazole core interacted with TYR283 (Figures S1 and S7–S10). The elagolix compound experienced conformational changes at its carboxylic acid end, interacting with ARG38. However, this end exhibited a higher degree of flexibility than that of the molecule coded 3665. While the critical protein-ligand contacts in elagolix were retained in the molecule coded 3665, they displayed differing bonding energies. In essence, the molecule coded 3665 preserved the fundamental protein-ligand interactions of elagolix, albeit with variations in their energetic characteristics (Figure S11).

The flexibility of the ligand binding site was remarkable in molecular dynamics simulations of the GnRH1R protein structure. The flexibility of the binding site was the biggest problem hindering the uniform movement of ligands. Long-term simulations were necessary to overcome this problem and obtain near-true data. For this purpose, the motion of the hit compound was examined in detail with 1000 ns long simulations.

#### 3.2.2. Protein root mean square deviation (RMSD) analysis

**Figure 8:**
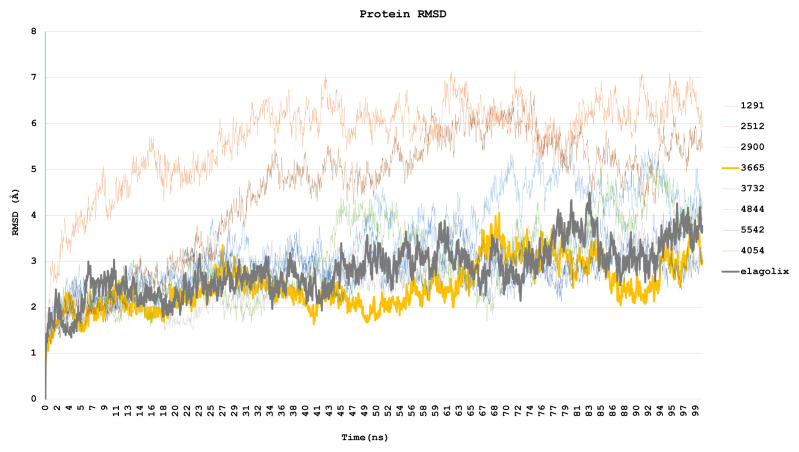
RMSD values to see the effect of ligands on the average positions of the GnRH1R protein structure throughout the molecular dynamics simulations.

The root mean square deviation (RMSD) measures the average change in displacement of a selection of atoms for a particular frame concerning a reference frame. Differences in RMSD values were used to monitor significant conformational changes in protein structure (Figure 8).

Protein structures observed to undergo visible conformational changes in the trajectory analysis were examined in detail in the RMSD plot. The mean RMSD value of the complex formed by the ligand coded 1291 was calculated as 3.499 Å, the average RMSD value of the complex formed by the ligand coded 2412 was calculated as 5.697 Å, and the mean RMSD value of the complex formed by the ligand coded 4054 was 4.499 Å.

The root-mean-square deviation (RMSD) values of the ligand coded 3665 and the complexes formed by elagolix exhibit an increasing trend. Despite the relatively low RMSD value of the ligand coded 3665, it was observed that it did not attain equilibrium. The same was noted for elagolix. It was found that these complexes did not induce significant conformational changes in the protein structure and had low RMSD values. Other ligands demonstrated equilibrium with the same RMSD values as elagolix.

Throughout the simulation, it was observed that the complex formed by the ligand coded 3665 displayed fluctuations in size. Specifically, the magnitude of the complex increased to 2.5 Å from 0 ns to 8.20 ns before decreasing to 2.0 Å by 16 ns. This fluctuation persisted until 67 ns when it briefly rose to 4 Å before rapidly declining to 2 Å by 88 ns. In addition, while the root-mean-square deviation (RMSD) value remained consistent up until 92 ns, it did experience a subsequent increase to 4 Å in the final stages of the simulation. Despite this, the RMSD values were unable to reach a plateau.

Likewise, the complex formed by elagolix displayed a trend toward increasing fluctuations throughout the simulation. The distances between the elagolix ligand and the GnRH1R receptor fluctuated between 1.8 Å and 4.3 Å, prompting a long-term simulation to gain further insight into this phenomenon.

Upon examination of the RMSD values of the elagolix and ligand 3665 proteins, it has been determined that the latter exhibits a lower value. This suggests that the 3665 ligands may have a more substantial antagonistic effect on GnRH1R than elagolix. This can be attributed to the 3665 ligand’s ability to effectively impede the GnRH1R protein structure movement.

#### 3.2.3. Protein root mean square fluctuation (RMSF) analysis

**Figure 9:**
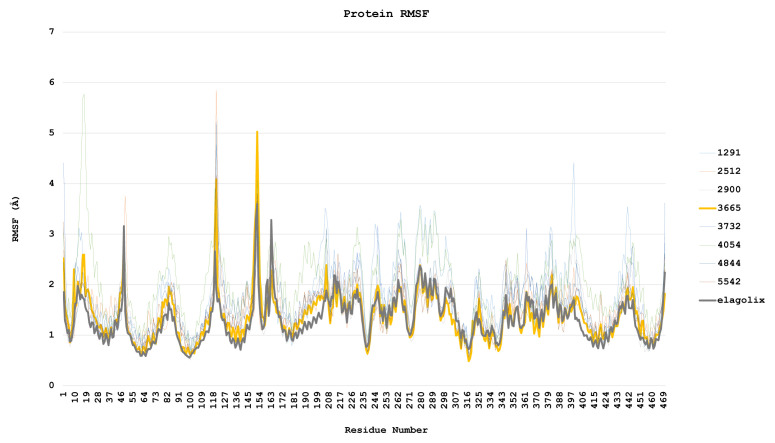
Protein RMSF plots, providing detailed insights into the fluctuations caused by ligands.

The study investigated how different ligands, including Elogolix, affect the dynamics of backbone atoms in the GnRH1 receptor structure. The root means square fluctuation (RMSF) values for backbone atoms were calculated, with higher RMSF values indicating greater flexibility of the protein regions. The most flexible areas were outside the membrane, which means the receptor’s signal transduction and proper function. The RMSF values of residues 222–240, 270–280, 311–324, 46–62, and 78–90 were much lower in the complexes formed by elagolix and 3665 compared to other ligands, indicating strong binding to the protein structure (Figure 9). Other ligands caused significant fluctuations at every graph point, suggesting weaker binding.

The ligand coded 3665 induced high flexibility in residues 121 and 151 of the GnRH1R protein structure, compared to elagolix. The secondary structure graphs showed that the widest gap between secondary structures had the highest fluctuations in both complexes. However, the 3665-GnRH1R complex had a single peak, indicating stronger binding and prevention of fluctuation.

### 3.3. MM-GBSA

The MM-GBSA calculation, which allows us to know the stability of the ligand-protein complex, is given in Table 2. preMM-GBSA is the binding energy of molecules at 0 ns. It is the free binding energy 100 ns after the start of the PostMM-GBSA molecular dynamics simulation. For example, compound 3665 has an energy value of −80.62 kcal/mol. However, the R form of the same compound has much higher energy (−52.68 kcal/mol). This demonstrates the effect of the same compound on the stability of the complex between the R and S forms.

The compound with the lowest binding energy was elagolix. Having a free binding energy of −102.32 kcal/mol, elagolix forms a more stable complex than all the compounds calculated for molecular docking.

It is seen that the complex formed by the Xanthinol compound, which achieves a much higher docking score than other compounds, is relatively stable with −73.37 kcal/mol. However, this compound formed a more unstable complex after 100 ns of simulation. On the other hand, the complex formed by the candesartan O-beta-D-glucuronoside compound is in a very stable state with an energy of −74.22 kcal/mol, like the candesartan N2-S-glucuronide compound. It was determined that the candesartan N2-S-glucuronide compound, which gave the best result among the docking scores, was the most stable among the complexes with an energy of −80.62 kcal/mol. The stabilities of the compounds differed enantioselectively within the protein structure. As a result, the R form of the compound coded 3665, the compound coded 3732, has higher energy.

After analyzing the data, it has been observed that the Gemcitabine compound obtained the lowest docking score and consequently showed the weakest outcome in the MM-GBSA calculation. This outcome implies that the Gemcitabine compound’s complex is relatively less stable when compared to other compounds.

When evaluated in general, Table 2 shows that the complexes formed by candesartan and its derivatives with the GnRH1R protein structure are relatively stable. It is seen that the inosine compound, like the Gemcitabine compound, has high energy and is unstable compared to other compounds. The MM-GBSA energy of the candesartan compound decreased in the 100 ns molecular dynamics simulations. There was no appreciable change in the energy of the elagolix compound, and it constitutes by far the lowest energy complex.

### 3.4. Long-term molecular dynamics simulations

MM-GBSA calculations of elagolix and 3665-encoded ligand and molecular dynamics simulation data gave far the best results from other compounds. To evaluate the potential of the compound coded 3665 as a good GnRH1R antagonist as the cognate ligand elagolix, long-term molecular dynamics simulations were performed, and MM-GBSA calculations were performed. To understand the effects of these two ligands on the GnRH1R protein structure, protein RMSD (Figure S12) values and protein RMSF (Figure S13) values of the two ligands were examined in detail during 1000 ns simulation.

The 3665-encoded ligand interacted with residue TYR283 during molecular dynamics simulation. In particular, there was a pi-pi interaction with the tetrazole ring. In addition to the interaction with the tetrazole ring, the ring of the biphenyl rings in the molecule, which is close to the benzimidazole group, fixes the center of the molecule to the TYR283 residue and preserves these effects during the simulation. Interaction with the carboxylic acid group attached to the benzimidazole group supports this effect. This effect continues throughout the simulation. Starting from 36 ns, the ARG38 residue, known to have an essential place in GnRH1R activity, and the carbonyl oxygen in the glucuronide group made hydrogen bonds. The strength of this interaction (shortening of the bond) increased with the change of conformation of residue ARG38, salt bridge, and extra hydrogen bonds were formed. The bridges constructed by the ASN27 residue over water molecules have carbonyl oxygen attached to the benzimidazole group in the ligand and the hydroxyl groups in the glucuronide group. These water molecules act in most of the simulation, positioning the conformational integrity of the ligand concerning the protein structure.

Regarding 258 ns in the simulation, the pi-pi interactions established with the TYR283 residue further increased their effect and number to four. The carbonyl oxygen at residue TYR283 is also involved in these pi-pi interactions. After 464 ns, the carboxylic acid group attached to the benzimidazole group made a 120-degree rotation. As a result of this rotation, the hydrogen bonds with the residue TYR283 were broken. Consequently, new hydrogen bonds are formed with the oxygen of the carboxylic acid group. Interactions with residue TYR283 continued through the oxygen atom. At 511 ns, a 70-degree turn occurred in the ring of the biphenyl group close to the tetrazole. This rotation movement caused the pi-pi interaction of this right group and the residue TRY283 to break. The tetrazole ring of residue TYR283 and the pi-pi interaction in the imidazole ring in the benzimidazole group continued. With this rotation, a new hydrogen bond was formed with the OH group in the TYR283 residue and the nitrogen atom in the imidazole part of the benzimidazole group. After 524 ns, the benzimidazole group underwent a 90-degree rotation, which moved the carbonyl group in the benzimidazole ring away from the TYR283 residue, but the pi-pi interactions continued. Based on the latest observation, the benzimidazole group underwent its final rotation within 525 ns, establishing an additional hydrogen bond with the residue ASN27. For a more detailed analysis of the distances between critical interactions, please refer to the supporting material (Figures S1, S7, and S9).

As a result of strong intramolecular hydrogen bonding, the elagolix compound displays a more inflexible structure compared to the 3665-encoded ligand. The molecule strongly interacts with vital residues such as ASN27, TYR283, and LYS121, creating pi-pi interactions with TYR283. The anchoring of the molecule to the protein structure guarantees that the functional groups linked to the pyrimidine ring maintain their interactions.

The elagolix compound exhibits a distinctive feature that sets it apart from the ligand coded 3665, which is its interaction with the hydrophobic pocket and the formation of strong hydrogen bonds with the crucial ARG38 residue, essential for the function of the GnRH1R protein structure. The elagolix compound’s structure remained firm during the simulation, resulting in limited movement.

At the end of the 1000 ns simulation time, MM-GBSA calculations were conducted for elagolix and the ligand coded 3665. Elagolix had an MM-GBSA of −110.68 kcal/mol. The ligand coded 3665 had an MM-GBSA of −88.03 kcal/mol. These values indicate that the elagolix-GnRH1R complex was more stable at the end of the 1000 ns molecular dynamics simulation.

**Figure 10:**
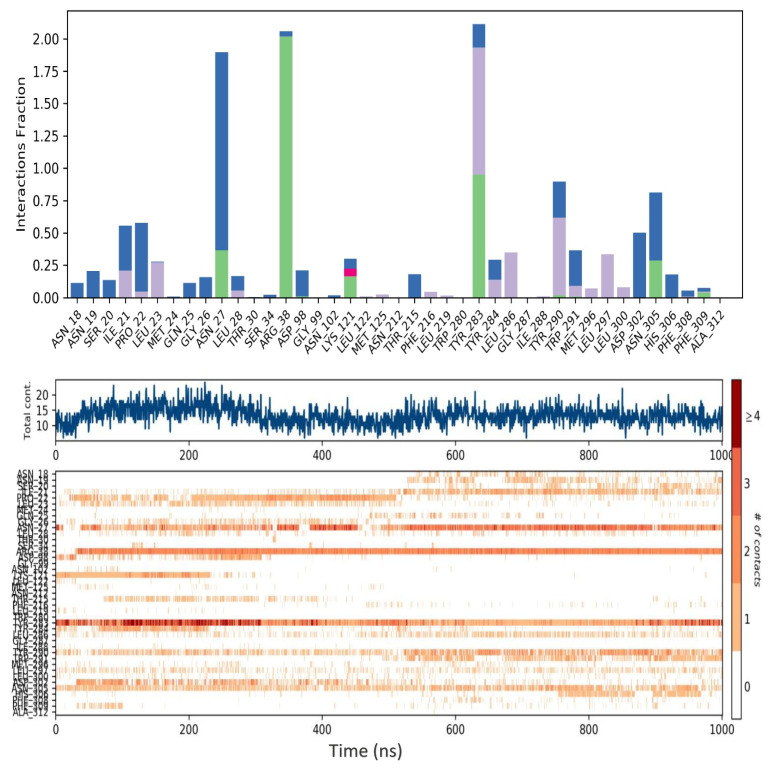
Contacts of ligands coded 3665 with amino acid residues during 1000-ns molecular dynamics simulation.

**Figure 11:**
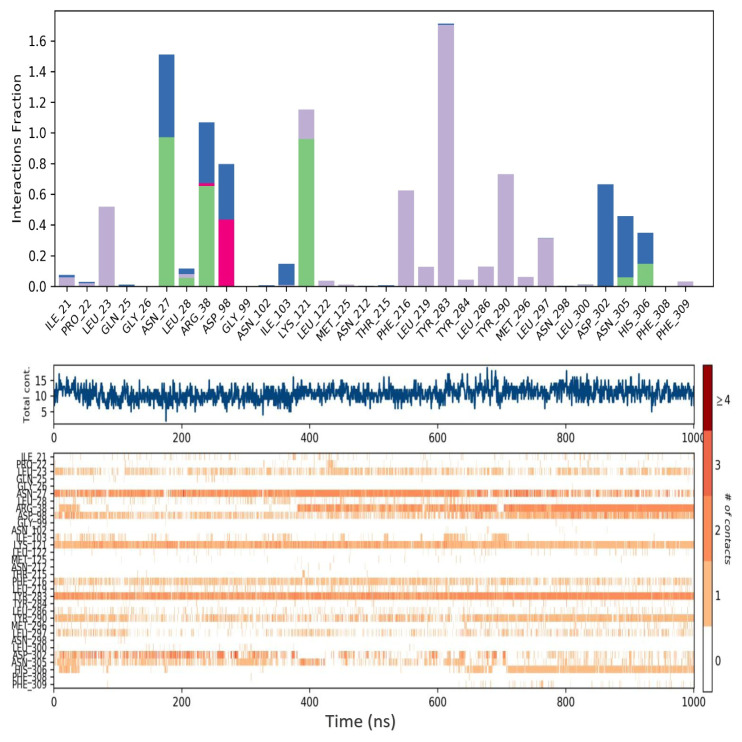
Contacts of elagolix ligand with amino acid residues during 1000 ns molecular dynamics simulation.

The number of interactions of the ligand coded 3665 with the GnRH1R protein structure is higher than that of the elagolix molecule with the GnRH1R protein structure (Figures 10 and 11). The molecular dynamics simulation of the ligand coded 3665 has revealed that its structural flexibility is the key driver for its mobility and fluctuations, particularly in the benzimidazole group, as evidenced in Figure S14. This has significant implications for the activity of the GnRH1R protein construct. The ligand also interacts with crucial amino acid residues beyond its initial binding site, providing critical data for long-term interactions.

For example, the elagolix compound exhibits prominent hydrophobic interactions, and the ligand coded 3665 forms strong hydrogen bonds with residue ARG38, as depicted in Figure S8. Furthermore, the ligand coded 3665 demonstrates higher total contact, especially in the first 400 ns of the simulation. These findings provide valuable insights into the complex molecular interactions of the ligand coded 3665 and its potential impact on the activity of the GnRH1R protein construct. Hydrogen bonding and hydrophobic interactions exist with the crucial amino acid residue, designated as TYR283, while the elagolix compound only experiences hydrophobic interactions with this residue. Another significant residue in the binding site is ASN305, with both compounds interacting with this residue.

GnRH analogs are widely employed to treat hormone-dependent diseases by inhibiting gonadotropin and sex steroid hormones [48]. The binding modes of GnRH analogs to the GnRH1R protein structure are referenced in developing new antagonist compounds. The GnRH binding residues (i.e., ASP98, ASN102, ASN212, TRP280, TRP289, and TYR290) of mammalian GnRH1R are highly conserved among vertebrate GnRHRs [51]. This site is also close in space to LYS121, located in the extracellular third of transmembrane helix 3, which is a likely contact site for agonists but not antagonists [52]. Evidence suggests that the ARG38 residue plays an essential role in stabilizing the active conformation of the receptor by forming a set of inter- and intramolecular interactions [53]. It revealed that ARG8 of GnRH I made contact with ASP302 while inhibiting cell growth. These findings provide the basis for developing selective GnRH analog cancer therapeutics that directly target tumor cells, inhibit pituitary gonadotropins, or both [48]. Interaction with these residues will impair the signal transmission of the GnRH1R protein structure and provide an antagonistic effect. Figures 10 and 11 provide a comprehensive account of the interactions between elagolix and ligands coded 3665 during the molecular dynamics simulation.

Molecular docking and molecular dynamics studies of many drug compounds, likely to have antagonistic effects in the GnRH1R protein structure, were carried out, and the stability of the complexes was investigated through MM-GBSA calculations. Detection of critical amino acid residues was determined using the cognate ligand elagolix and the literature. Furthermore, interactions with key amino acid residues were considered in the molecular docking study. Accordingly, residues TYR283, TYR290, LYS121, ASP98, and ASN305 were determined as key amino acid residues. The literature supported these residues, and residues that should interact with an antagonist ligand were determined [48,50–52]. In addition, high affinity for the binding site was evaluated between ligands docking to the receptor structure [29,52,54].

In the molecular docking study, eight ligands were identified as prominent, most of which were derivatives and enantiomers of the candesartan compound. Despite structural differences, all of these ligands shared everyday interactions with the TYR283 residue, which ensured the fixation of the molecule’s center to the protein structure. Other interactions, such as those with the LYS121 residue, strengthened their affinity for the receptor protein structure. The elagolix compound contained a hydrophilic pocket suitable for the high-affinity ligands, including interactions with the ASN27 residue, which were not present in the elagolix compound. Molecular dynamics studies showed that interactions with the ASN27 residue had an antagonistic effect. This residue was observed to have long-term interactions with the relevant ligands in all molecular dynamics studies.

In short molecular dynamics studies lasting 100 ns, eight ligands were screened, and a comparison was made between their data and that of elagolix. The comparison was based on the RMSD values, which indicate the extent to which the signal transmission of the GnRH1R construct is inhibited. The inhibition of conformational change of the GnRH1R protein structure is a sought-after feature in antagonist ligands, as it prevents the production and transmission of signals. The ligand coded 3665 was found to have very similar RMSD data to elagolix in 100 ns simulations, with minimal deviation in its mean position from the beginning to the end of the simulation.

The RMSF value was used to detect local fluctuations in protein structure. As expected, areas outside the membrane portion in the RMSF plot showed high fluctuations for all ligands (Figure 9). To interpret the fluctuation as signal transmission and production, fluctuations must be observed in the structures within the membrane. While such fluctuations of other ligands are at high levels, it is seen that they produce and transmit signals in parallel with the RMSD values. In contrast, ligand coded 3665 and elagolix differ from other ligands with low fluctuation in each region. In Figure 9, when the RMSF graphs are placed side by side and the GnRH1R structure is superimposed on the strands graph, it is determined that the last strand structure of the ligand coded 3665 is disrupted. Based on the observations, it appears that the deterioration also impacts signal generation. Figure S15 emphasizes that the degradation in the strand structure is particularly pronounced [55].

The RMSD values of the ligands, which denote the average deviation of ligands from the first clamping point, are observed to be at low levels for all ligands. The molecular dynamics simulation reveals that all ligands have primarily maintained their initial docking position, as depicted in Figure 8.

When all these data are evaluated, it is seen that the ligand coded 3665 is the most suitable candidate for comparison with elagolix. Therefore, this framework made 1000-ns-long molecular dynamics simulations of these two ligands.

During the 1000-ns molecular dynamics simulation, an interesting observation was made regarding the interaction between the TYR283 residue and the central region of the molecules. This observation is indicative of inhibition capacities. Elagolix, compared to the ligand coded 3665, exhibited weaker hydrophobic interactions with TYR283. However, it demonstrated stronger hydrogen bonds (Figure S16), water bridges, and hydrophobic interactions. The interaction with ARG38 was observed throughout the simulation in both compounds, indicating the antagonistic effect of the ligand coded 3665. While the elagolix molecule interacted with ARG38 after 400 ns, the ligand coded 3665 interacted after 70 ns and sustained the interaction throughout the simulation. Lastly, elagolix exhibited higher interactions with the ASP98 residue at the ligand binding site in the GnRH1R structure than the ligand coded 3665.

The objective was to predict the stability of the protein-ligand complex through MM-GBSA calculations. The calculations were performed at the initial docking stage and the end of a 100-ns molecular dynamics simulation for each ligand. Results showed that candesartan and its derivatives dissociated from other ligands, and the ligands coded 3665 and 3723, which are enantiomers, had significantly different energy values (Table 2). Elagolix had the best energy values, indicating the most stable complex that remained stable over time. The MM-GBSA value at the end of a 1000-ns molecular dynamics simulation showed little change, meaning that elagolix and the ligand coded 3665 maintained their stability. Furthermore, the elagolix complex was more stable than the ligand coded 3665, which can be attributed to the former’s rigid structure. This analysis was supported by the MM-GBSA data for a similar molecule, the more rigid ligand coded 2900 (candesartan), at the end of a 100-ns molecular dynamics simulation (Table 2).

The elagolix molecule is notable for its unique intramolecular hydrogen bond, which enables it to maintain continuous interaction throughout the simulation period. This characteristic increases rigidity, making it highly effective in the GnRH1R structure. However, despite its rigidity, the molecule still faces a significant obstacle in drug design due to the flexibility of the binding site. Additional information regarding this topic can be found in the supporting material (Figure S17). The supporting material (Figures S2, S8, and S10) also demonstrates the impact of ligands on the GnRH1R structure, which contains numerous mobile elements in the binding region. While the rigid elagolix molecule cannot influence the strand structures, the flexibility of the ligand coded 3665 disrupts the strand structure and inhibits signal transmission. This evidence suggests that the ligand coded 3665 may be a superior antagonist to elagolix.

The results of a 1000-ns molecular dynamics simulation show a significant similarity between the protein RMSD data of two ligands, suggesting that the ligand coded 3665 may have similar effectiveness to elagolix. This is particularly evident when analyzing the RMSF graphs, as illustrated in Figure S15. Furthermore, upon closer examination, the 1000 ns simulation reveals that the fluctuations and fading of the ligands coded 3665 are akin to those of elagolix. These findings suggest that both compounds will likely have identical binding modes in the GnRH1R protein structure and may have similar effects (Figure S15)

### 3.5. Movement and conformation analysis of protein structures

**Figure 12:**
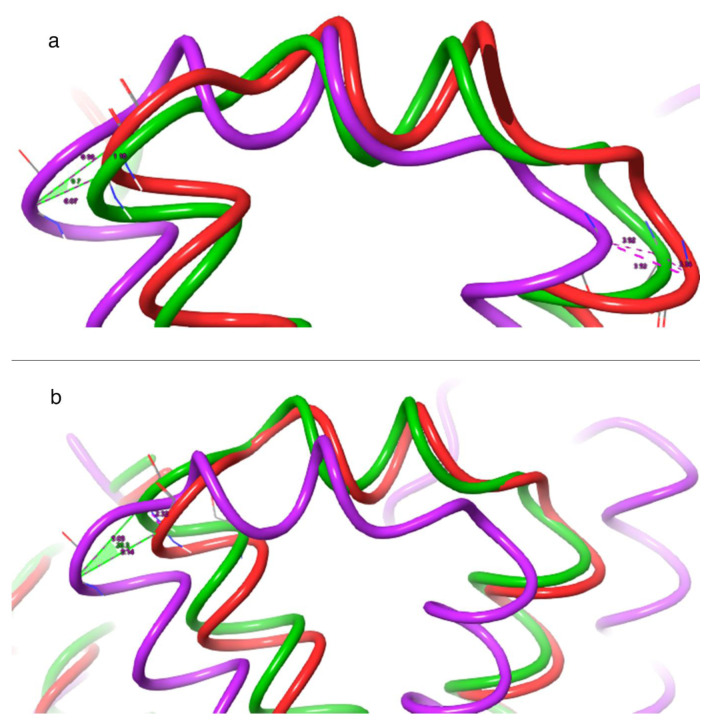
Important movements of protein structures: (a) Movement caused by 3665 ligand; (b) Movement caused by elagolix ligand. Purple color at 0 ns, green at 500 ns, and red at 1000 ns.

For the analysis of the protein structures, long-term molecular dynamics simulations of the structures containing the ligand coded 3665 identified as the lead compound and the cognate ligand elagolix were used. The effects of these ligands on protein structure were analyzed in detail and compared with each other. Figure 12 shows the conformational differentiation of the previously mentioned critical residues and other surrounding residues. Structures with the same conformation at 0 ns (tube structures shown in purple in Figure 12) have very different conformations after 500 ns. After 500 ns, the movement caused by the ligand coded 3665 is about 6 Å, while the position difference between the two times caused by the elagolix compound contains a difference of about 9 Å. The difference in position caused by the two ligands reveals that the ligand coded 3665 brings about a lesser displacement than elagolix. The ligand coded 3665, which caused a minor difference, imposed constraints on the movement of the protein structure and repressed signaling more effectively than elagolix. The angle formed between the positions of the protein structures at three-time intervals provides precise information about the displacement. A 9-degree interval occurs between the time-varying positions of the ligand coded 3665, while a 20-degree interval occurs between the positions of the movements caused by the elagolix compound. The difference between the time-varying positions caused by the ligand coded 3665 is significantly smaller than that of the elagolix, which is substantial evidence that the movement of the protein structure is limited and signal transmission is blocked. The apparent change in the switch structure in Figure 12 gives essential information about signal transmission. In the switch region, which consists of reversible structures between two or more molecules, the signal transmission area has become “closed” with 3665. In the elagolix-bound protein structure, the switch structure is more mobile and partially open.

**Figure 13:**
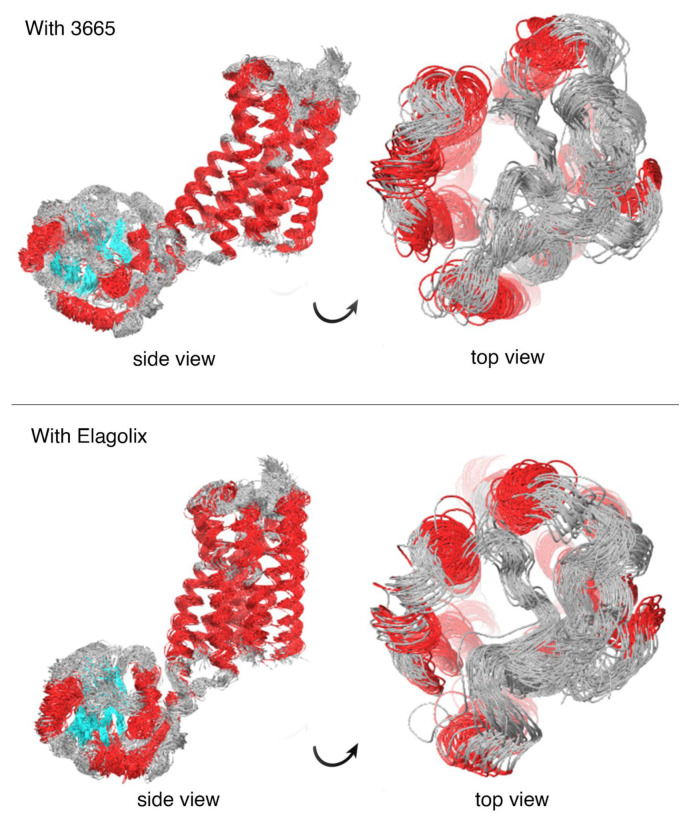
Superimposed images of structures recorded at 25 ns intervals of 1000 ns molecular dynamics simulations with elogolix and 3665 ligands bound to the GnRH1 receptor protein. Red areas are protein secondary structures. For clarity of visualization, the membrane structure is not shown.

In Figure 13, the overlay of protein structure images obtained from 1000-ns molecular dynamics simulations clearly illustrates the impact of elagolix and ligand coded 3665 on protein structures. The secondary structures, highlighted in red in Figure 13, indicate that the ligand coded 3665 significantly inhibits the signal transduction of the receptor protein compared to the elagolix.

The secondary structures of the ligand coded 3665 exhibit more significant disruption than elagolix. Molecular dynamics simulations reveal reduced detection of the latter, with the red areas being bound explicitly to the ligands coded 3665. Furthermore, considerable disruptions are observed in the region under the membrane structure, responsible for transmitting signal transduction to the cell’s internal environment when bound to the ligand coded 3665.

Distortions in the secondary structures are predominantly observed in the ligand-binding region. This suggests that binding a ligand coded 3665 induces conformational changes in the protein structure. When assessed with the changes in the area underneath the membrane structure, it poses a significant limitation in signal transduction.

**Figure 14:**
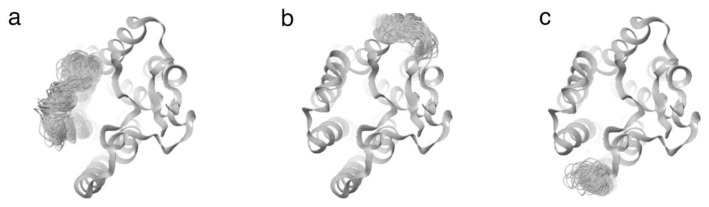
Collective representation of important movements in the GnRH1R-3665 complex structure on the ribbon image.

The ribbon visualization in Figure 14 displays the most dynamic parts of the GnRH1R-3665 complex during the molecular dynamics simulation. Specifically, the structure depicted in Figure 14a represents a protein component that behaves as a switch. The switch’s movement is observed in the image resulting from superimposing the molecular dynamics simulation images. During this process, the switch structure shifts outward from the protein structure’s center. This movement is essential in preventing signal transduction and acting as an antagonist of the ligand coded 3665. In Figure 14b, the movement of a part of the outline of the protein structure is given. It is seen that the movement of this part is limited. The transmission of the generated signal deep into the membrane structure is prevented by limiting the activity of this part. Figure 14c shows the fluctuation of one of the ends of the GnRH1R protein structure. While the fluctuation of this particular segment is not indicative of signal generation or transmission, it plays a crucial role in protein structure transduction. The segment in Figure 14c moves outward from the protein structure, which signals its detachment from the ligand effect.

The analysis of protein structure movement induced by ligands in Figures 13 and 14 reveal that the ligand coded 3665 significantly affects the free ends oscillating across the molecular dynamics simulation of the GnRH1R protein. The top view of Figure 13 shows that the free ends outside the membrane exhibit minimal fluctuation compared to the significant movement caused by the ligand coded 3665 on the protein structure, as opposed to the movement caused by elagolix. Since signal generation is facilitated by the movement of regions in protein structure, the ligand coded 3665 possesses a high potential to exhibit a better antagonist effect than elagolix.

## 4. Conclusion

Recent advancements in scientific research have enabled the utilization of advanced molecular docking and molecular dynamics simulation techniques to identify potential GnRH1R antagonists that could effectively treat uterine fibroids, prostate cancer, and endometriosis. The study involved screening 3447 drug compounds from the ZINC15 database using molecular docking techniques and employing MD simulation to validate the relative stability of the hit compounds. This study presents a promising computational drug reuse approach for discovering novel antagonist compounds for GnRH1R, with ligand 3665 emerging as a preclinical-early-stage candidate. After a thorough evaluation, the candesartan N2-S-glucuronide (ligand coded 3665) compound was identified as a potential GnRH1R antagonist. These findings indicate the potential of this approach for efficient drug discovery and development.

## Supporting Materials

Figure S1 The distance variation of the benzimidazole domain of the 3665 coded ligand from the Tyr283 residue over time during 1000 ns simulation.

Figure S2 The variation of the distance of the carbonyl group from the Lys121 residue in the elagolix molecule with time during the 1000 ns simulation period.

Figure S3 Cross-sectional view of the binding site of candesartan O-beta-D-glucuronoside compound.

Figure S4 Cross-sectional view of the binding site of candesartan compound.

Figure S5 Cross-sectional view of the binding site of candesartan N2-S-glucuronide compound.

Figure S6 Cross-sectional view of the binding site of candesartan N2-R-glucuronide compound.

Figure S7 The variation of the distance of the glucuronide group (1) of the 3665 coded ligand from the Arg38 residue during the 1000 ns simulation period (first interaction).

Figure S8 The variation of the distance of the carboxylic acid group of the elagolix ligand from the Arg38 residue during the 1000 ns simulation period (first interaction).

Figure S9 The variation of the distance of the glucuronide group (2) of the 3665 coded ligand from the Arg38 residue during the 1000 ns simulation period (second interaction).

Figure S10 The variation of the distance of the carboxylic acid group from the Lys27 residue in the elagolix molecule with time during the 1000 ns simulation period.

Figure S11 The variation of the number of interactions made by the ligand coded 3665 during the 1000 ns simulation.

Figure S12 Protein RMSD values in 1000-ns molecular dynamics simulation.

Figure S13 Protein RMSF values in 1000-ns molecular dynamics simulation.

Figure S14 RMSF values of ligand coded 3665 during 1000-ns simulation.

Figure S15 (a) RMSF values of a 3665-GnRH1R complex with seconder structure (light red), strands (light blue), and relationship of strands and protein structures dark blue line; (b) RMSF values of elagolix-GnRH1R complex with seconder structure (light red), strands (light blue), and relationship of strands and protein structures dark blue line.

Figure S16 Variation of the number of hydrogen bonds of the ligand coded 3655 during the 1000-ns simulation.

Figure S17 Variation of the number of intramolecular hydrogen bonds of elagolix and ligand coded 3665 with time.

## Figures and Tables

**Table 1:** Induced fit docking scores.

Code–molecule name	Molecule structure	IFD docking score (kcal/mol)	H-bond residue
1291-candesartan O-beta-D-glucuronoside	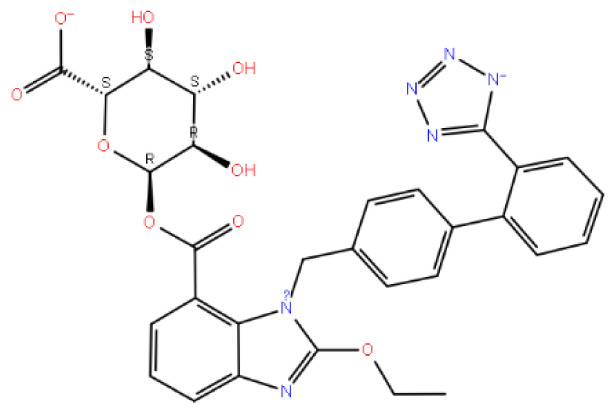	**−15.725**	TRY283, THR190, GLN208, ASN212, THR215, and ILE21
2512-inosine	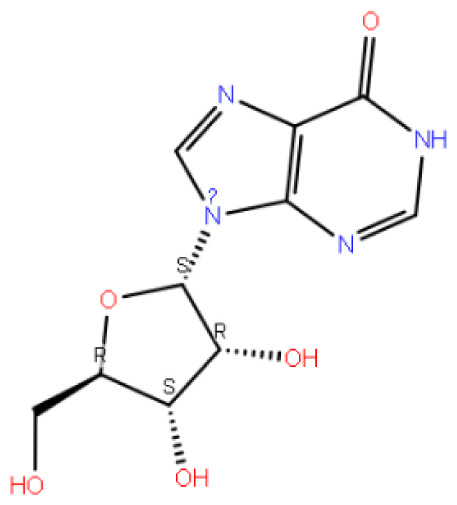	−9.030	ASN27, HIS306, THR42, and GLY99
2900-candesartan	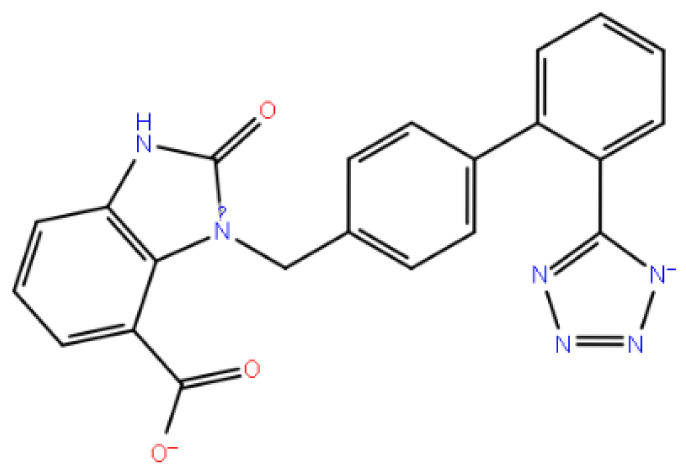	**−11.375**	LYS121 and TYR283
3665-candesartan N2-S-glucuronide	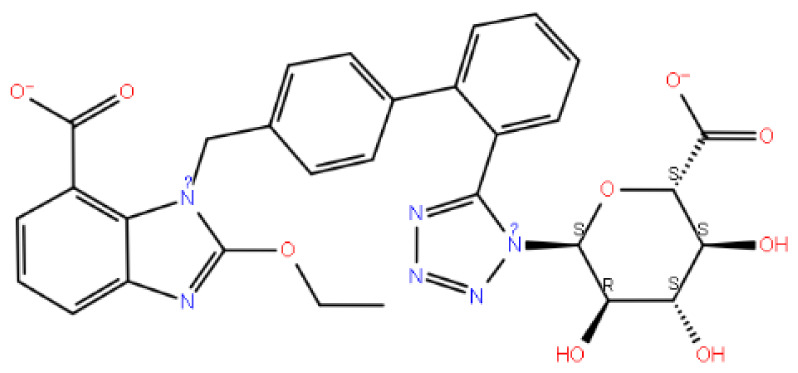	**−16.020**	PRO22, GLN25, ASN27, LYS121, and TYR283
3732-candesartan N2-R-glucuronide	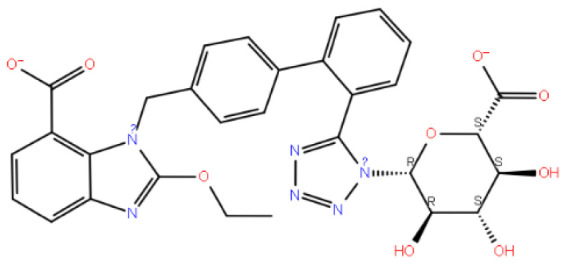	**−15.329**	LYS121, ASN212, and ASN27
4054-xanthinol	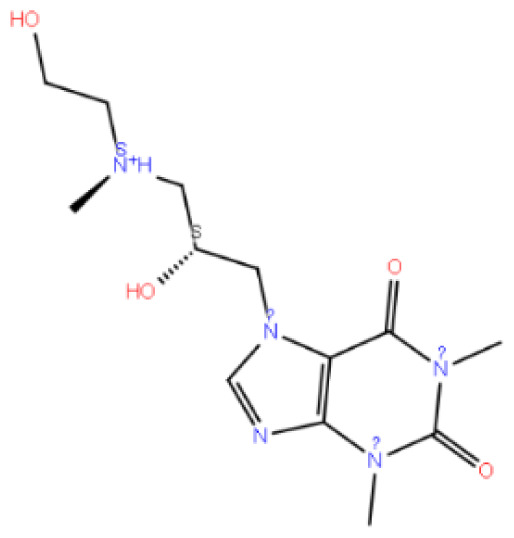	−8.446	ASN212 and TYR283
4844-gemcitabine	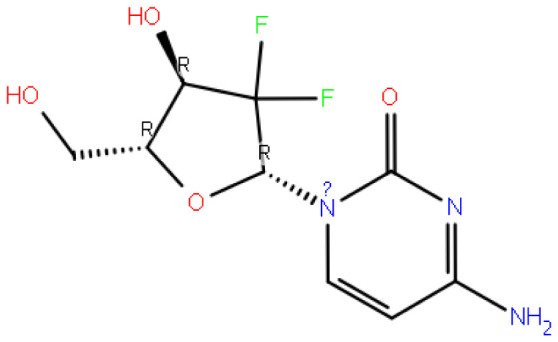	−7.401	PRO22, LYS121, and GLY287
5542-bortezomib	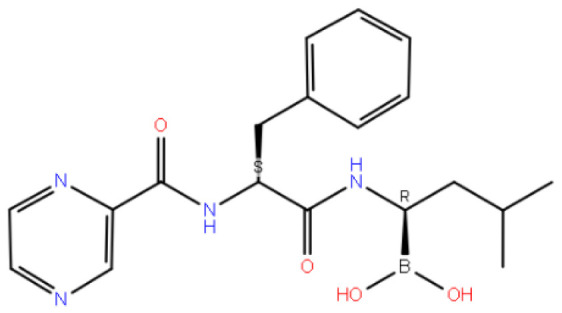	−12.109	ASN305

**Table 2:** MM-GBSA calculations for compounds.

Code–molecule name	preMM-GBSA (kcal/mol)- 0 ns	postMM-GBSA(kcal/mol) 100 ns

1291-candesartan O-beta-D-glucuronoside	−74.22	−61.17
2512 -inosine	−42.03	−63.50
2900-candesartan	−62.94	**−86.66**
3665-candesartan N2-S-glucuronide	**−80.62**	**−83.58**
3732-candesartan N2-R-glucuronide	−52.68	−55.20
4054-xanthinol	−73.37	−58.55
4844-gemcitabine	−41.67	−54.02
5542-bortezomib	−68.69	−69.38
Elagolix	**−102.32**	**−103.22**

## Footnotes

1 U.S. Food and Drug Administration [online]. https://www.fda.gov/consumers/knowledge-and-news-women-owh-blog/understanding-endometriosis-symptoms-treatment / [accessed 16 May 2023].

2 RCSB PDB. Protein Data bank [online]. https://www.rcsb.org/ [accessed 20 June 2022]

3 Schrödinger release 2023-1: Protein Preparation Wizard; Epik, Schrödinger, LLC, New York, NY, 2023.

4 Schrödinger Release 2023-1: Epik, Schrödinger, LLC, New York, NY, 2023.

5 Schrödinger Release 2023-1: LigPrep, Schrödinger, LLC, New York, NY, 2023.

6 Schrödinger Release 2023-1: Glide, Schrödinger, LLC,New York, NY, 2023.

7 Schrödinger Release 2023-1: Induced Fit Docking protocol; Glide, Schrödinger, LLC, New York, NY, 2023.

8 Schrödinger Release 2023-1: Desmond Molecular Dynamics System, D. E. Shaw Research, New York, NY, 2023.
