# Supplementary figures and images for: Computational drug repurposing effort for identifying novel hits for the treatment of diseases such as endometriosis, uterine fibroids, and prostate cancer
Source: Turk J Chem. 2024 Jan 4;48(2):402–21. doi: 10.55730/1300-0527.3667 (PMC11265929; doi:10.55730/1300-0527.3667)

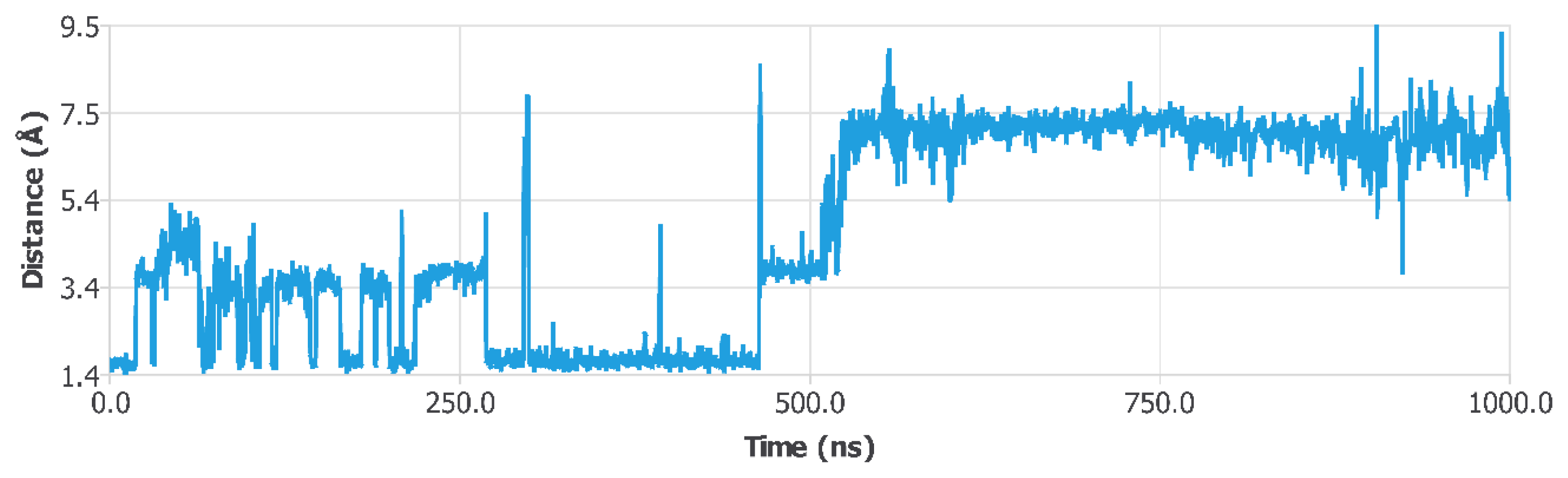

Supplement: Figure S1 — The distance variation of the benzimidazole domain of the 3665 coded ligand from the Tyr283 residue over time during 1000 ns simulation. [file tjc-48-02-402s1.tif]

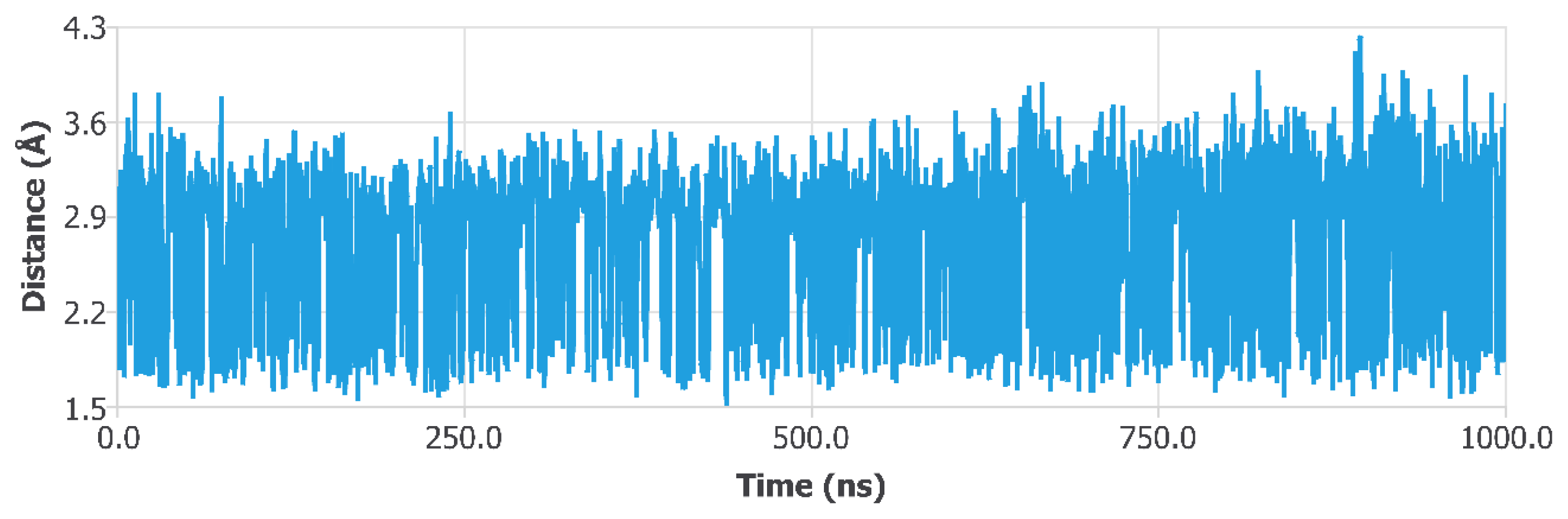

Supplement: Figure S2 — The variation of the distance of the carbonyl group from the Lys121 residue in the elagolix molecule with time during the 1000 ns simulation period. [file tjc-48-02-402s2.tif]

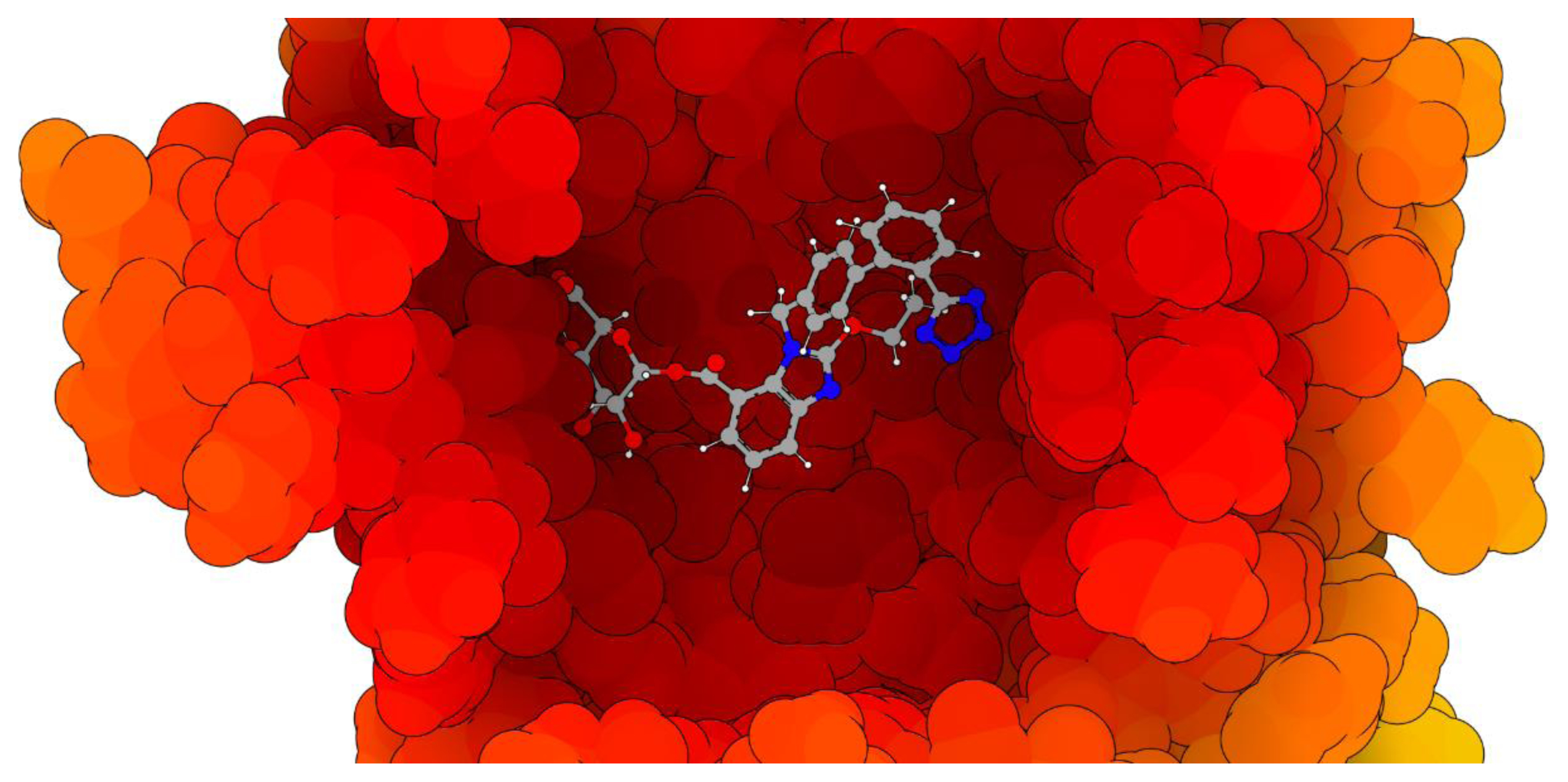

Supplement: Figure S3 — Cross-sectional view of the binding site of candesartan O-beta-D-glucuronoside compound. [file tjc-48-02-402s3.tif]

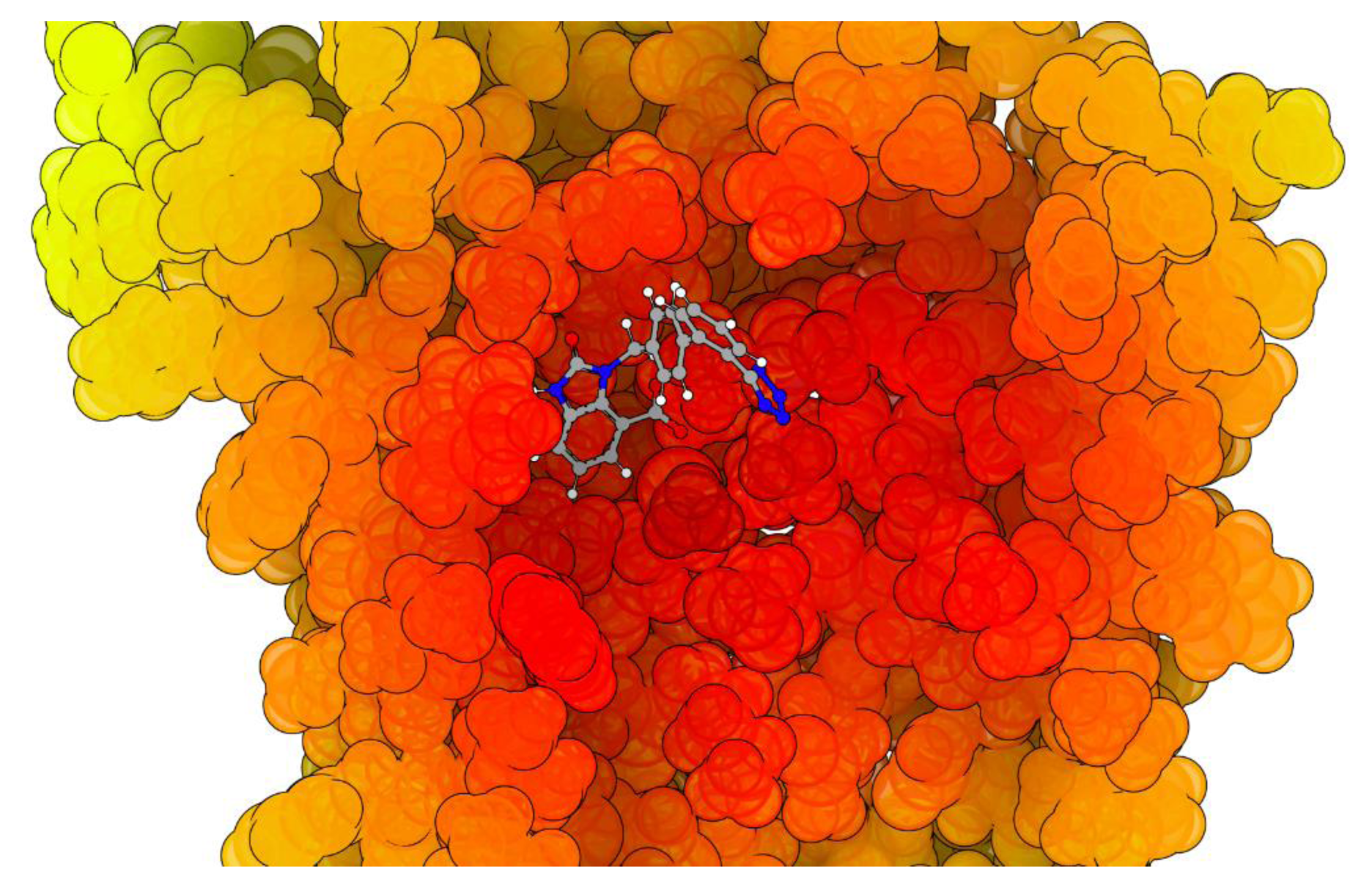

Supplement: Figure S4 — Cross-sectional view of the binding site of candesartan compound. [file tjc-48-02-402s4.tif]

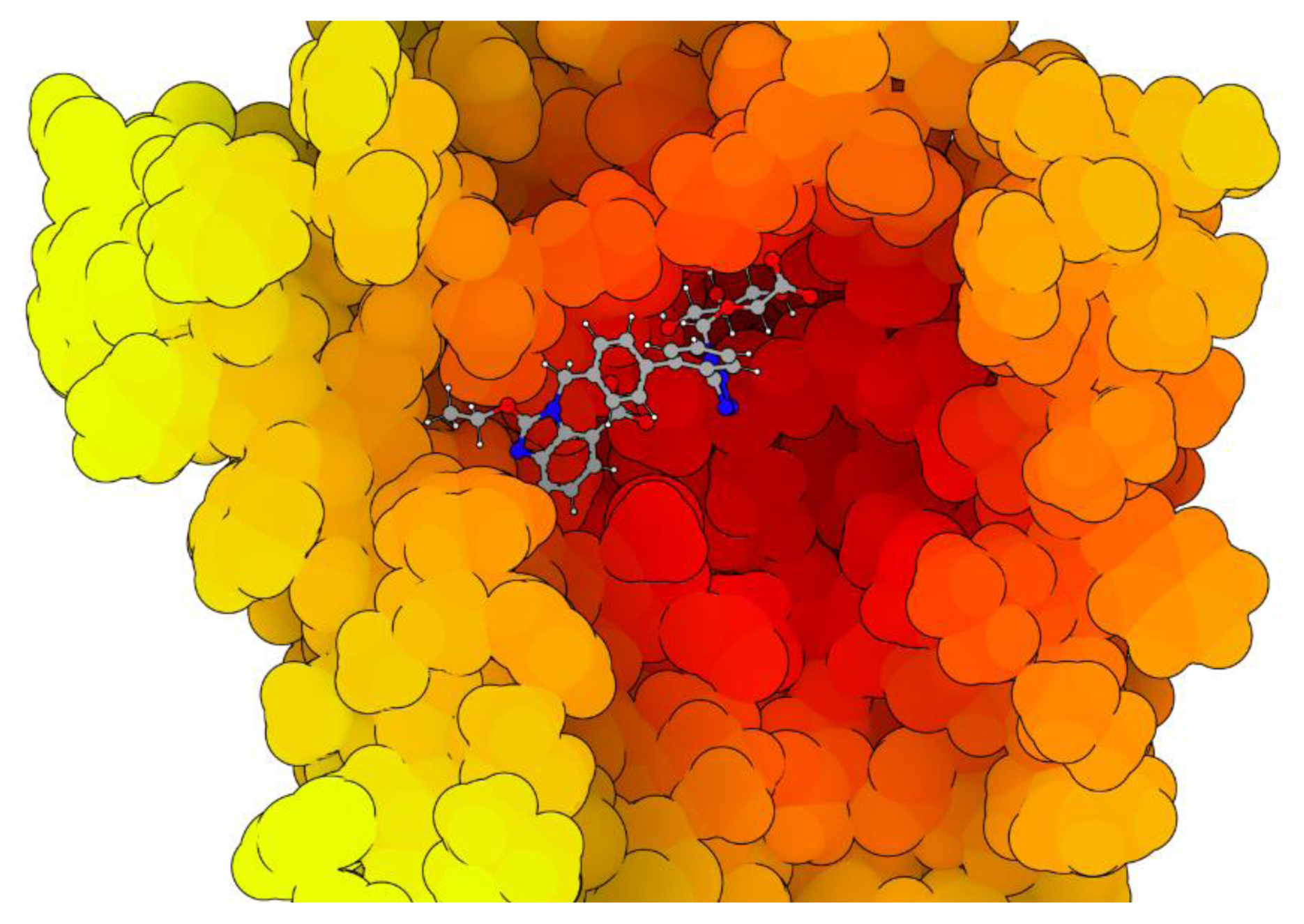

Supplement: Figure S5 — Cross-sectional view of the binding site of candesartan N2-S-glucuronide compound. [file tjc-48-02-402s5.tif]

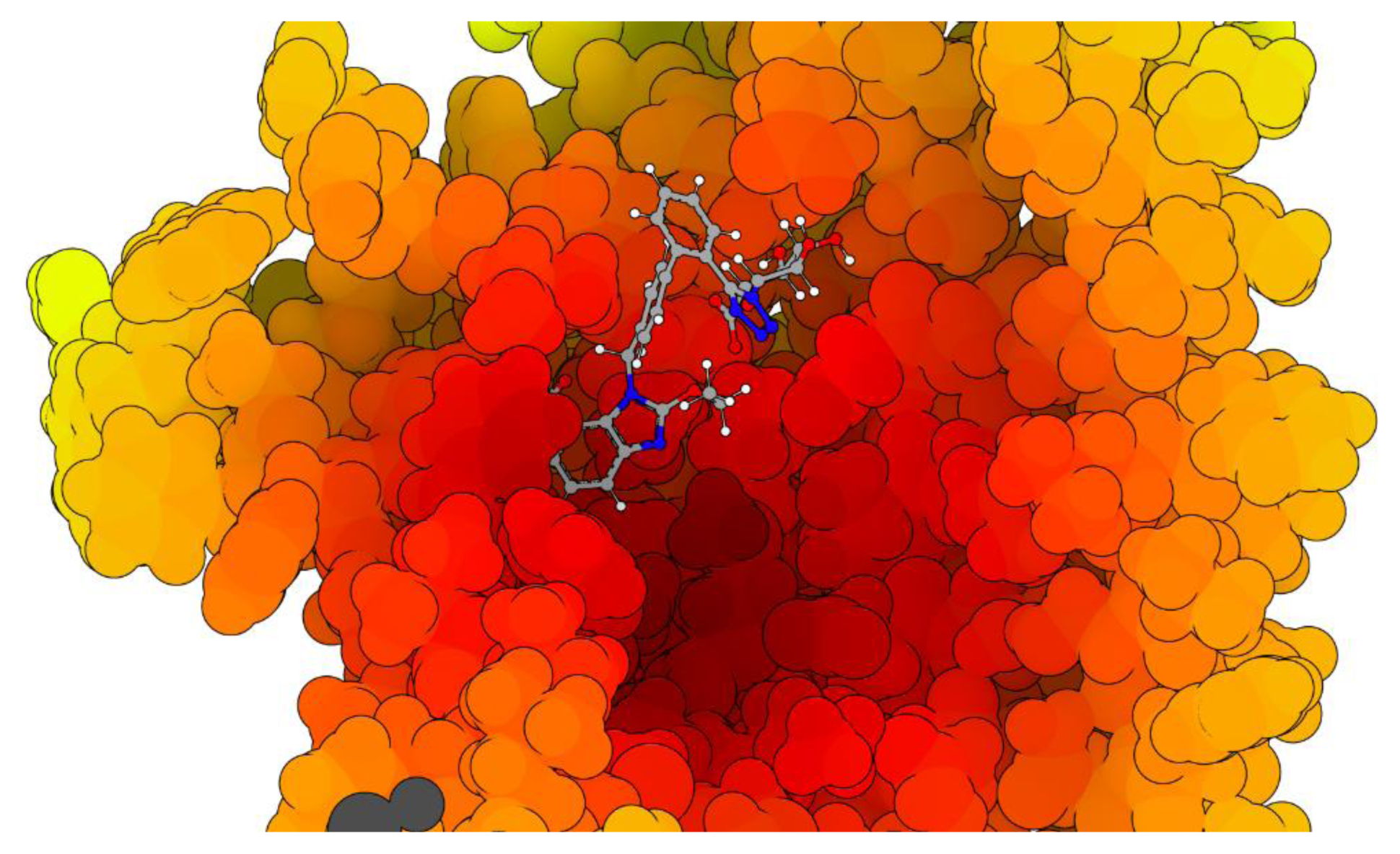

Supplement: Figure S6 — Cross-sectional view of the binding site of candesartan N2-R-glucuronide compound. [file tjc-48-02-402s6.tif]

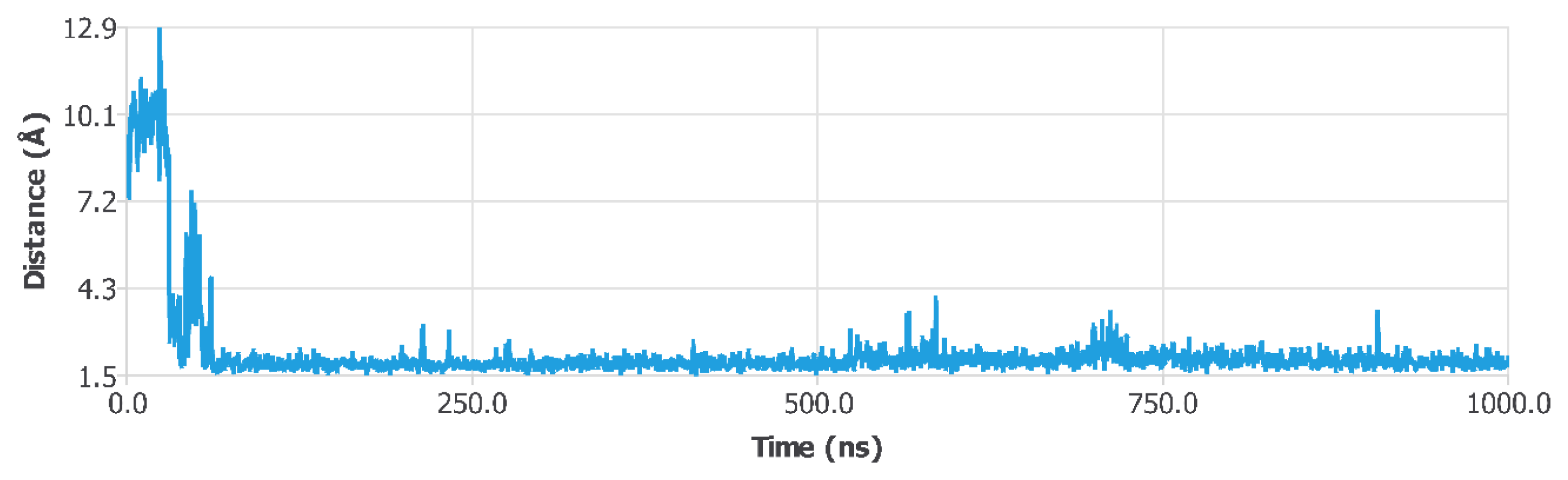

Supplement: Figure S7 — The variation of the distance of the glucuronide group (1) of the 3665 coded ligand from the Arg38 residue during the 1000 ns simulation period (first interaction). [file tjc-48-02-402s7.tif]

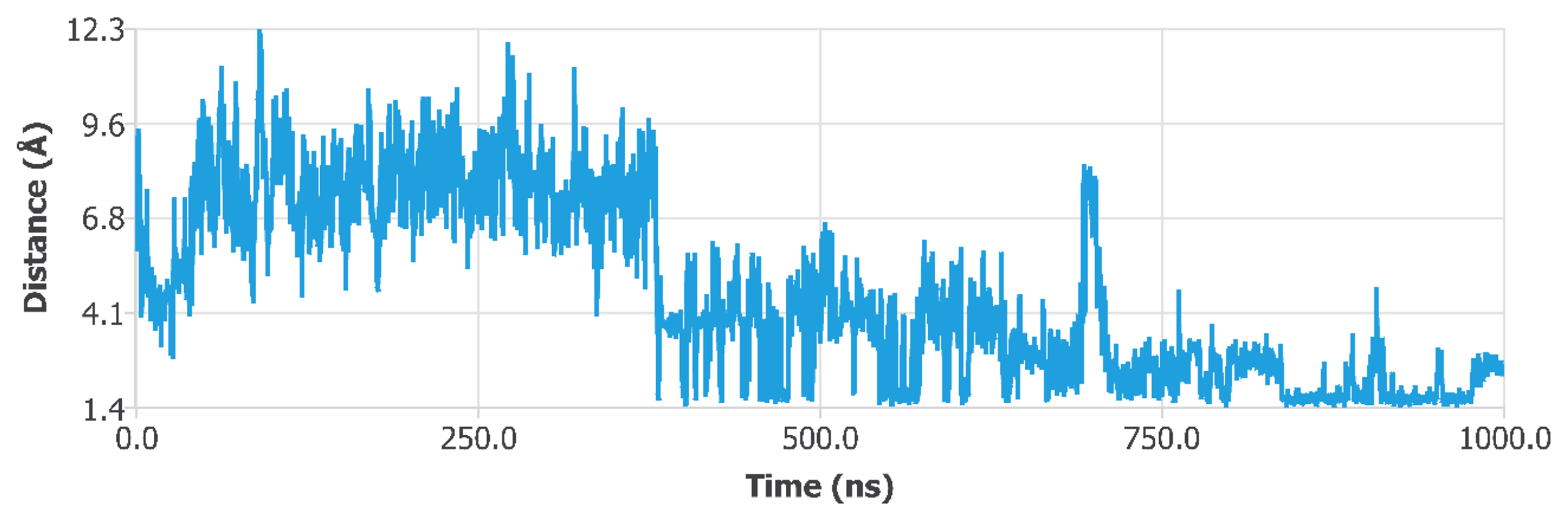

Supplement: Figure S8 — The variation of the distance of the carboxylic acid group of the elagolix ligand from the Arg38 residue during the 1000 ns simulation period (first interaction). [file tjc-48-02-402s8.tif]

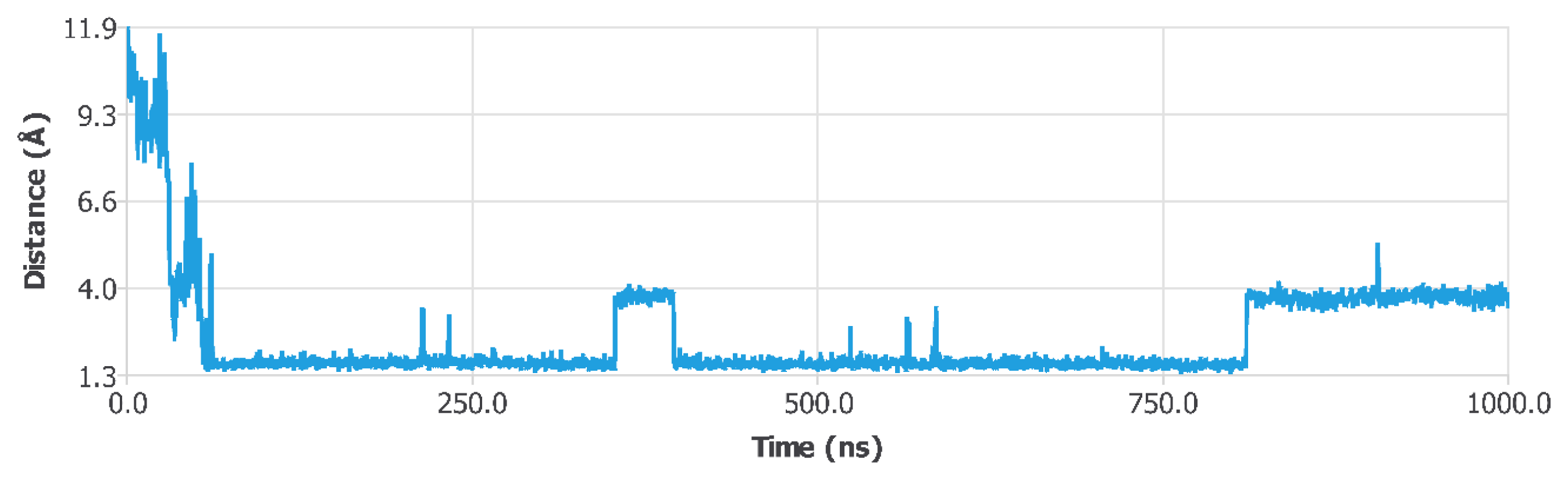

Supplement: Figure S9 — The variation of the distance of the glucuronide group (2) of the 3665 coded ligand from the Arg38 residue during the 1000 ns simulation period (second interaction). [file tjc-48-02-402s9.tif]

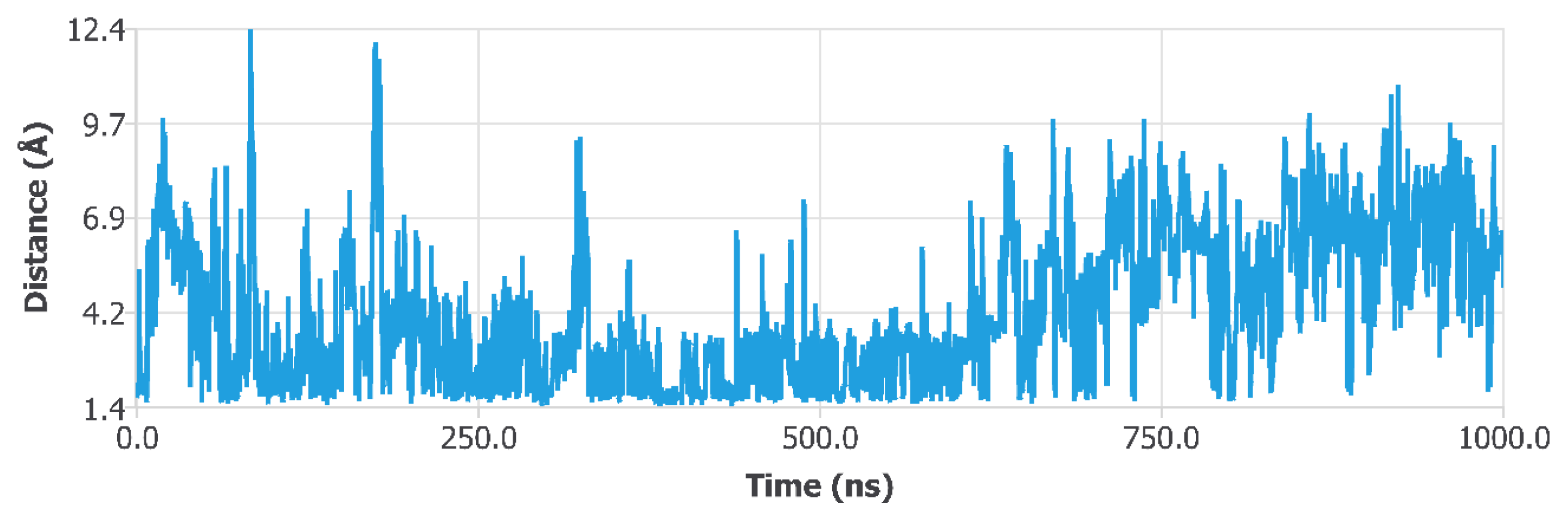

Supplement: Figure S10 — The variation of the distance of the carboxylic acid group from the Lys27 residue in the elagolix molecule with time during the 1000 ns simulation period. [file tjc-48-02-402s10.tif]

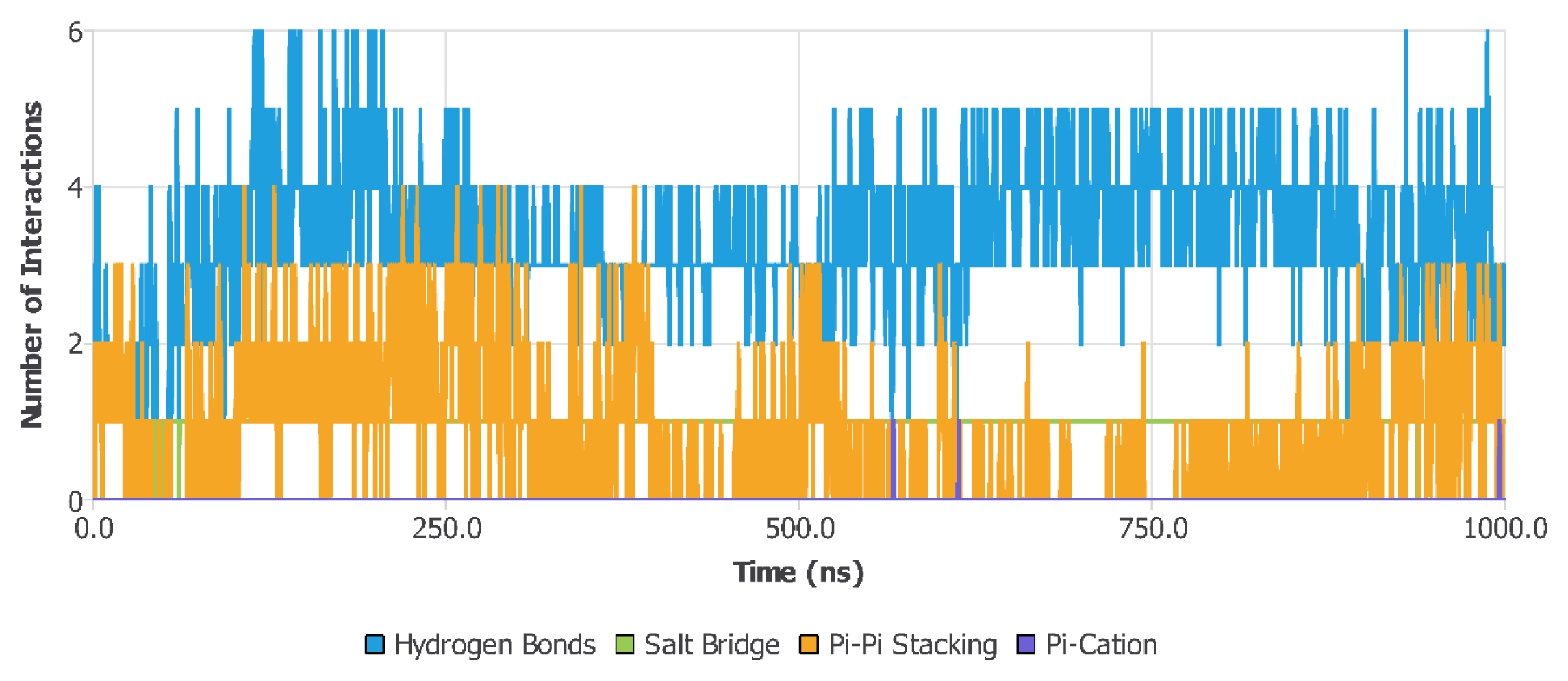

Supplement: Figure S11 — The variation of the number of interactions made by the ligand coded 3665 during the 1000 ns simulation. [file tjc-48-02-402s11.tif]

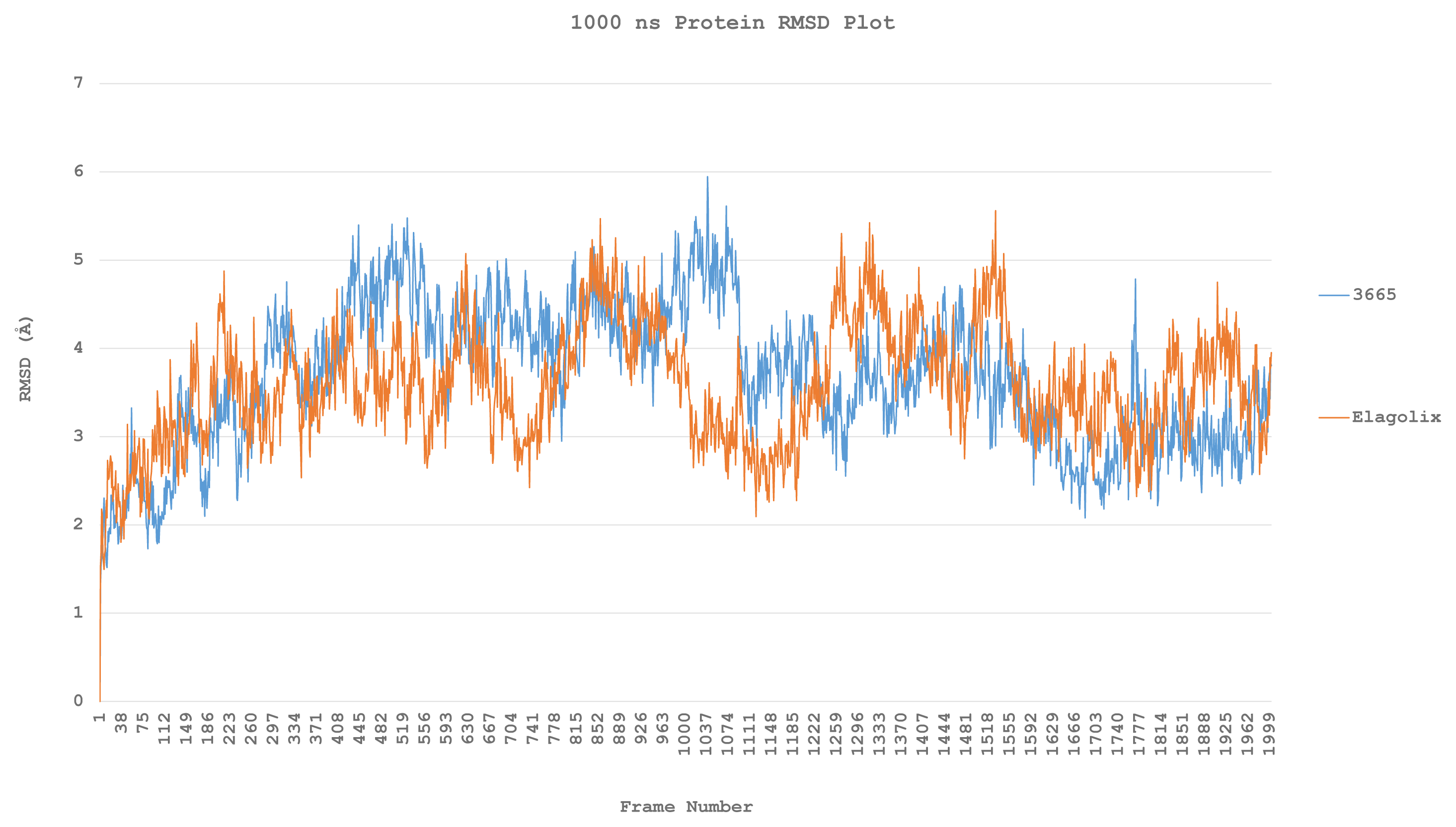

Supplement: Figure S12 — Protein RMSD values in 1000-ns molecular dynamics simulation. [file tjc-48-02-402s12.tif]

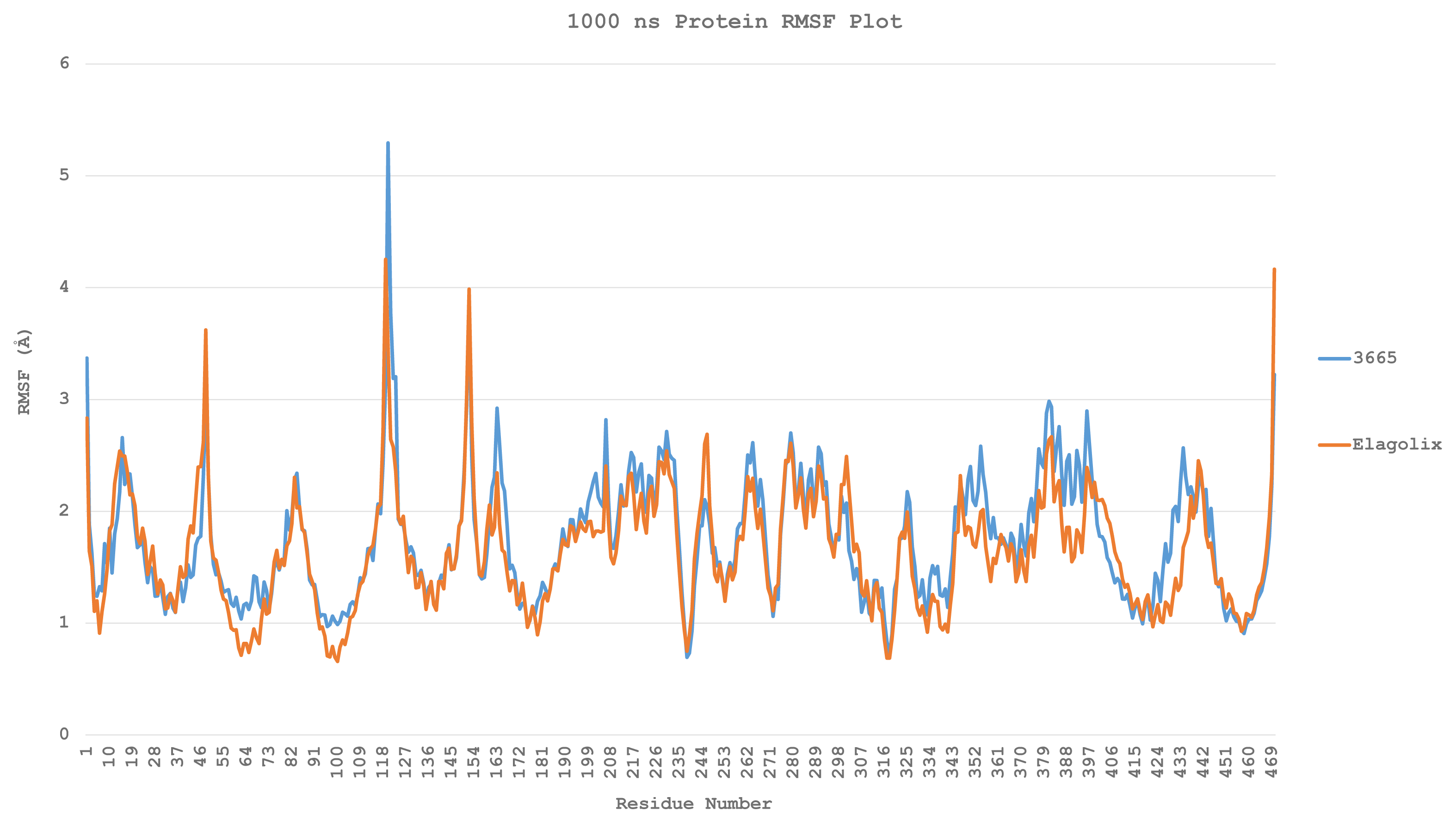

Supplement: Figure S13 — Protein RMSF values in 1000-ns molecular dynamics simulation. [file tjc-48-02-402s13.tif]

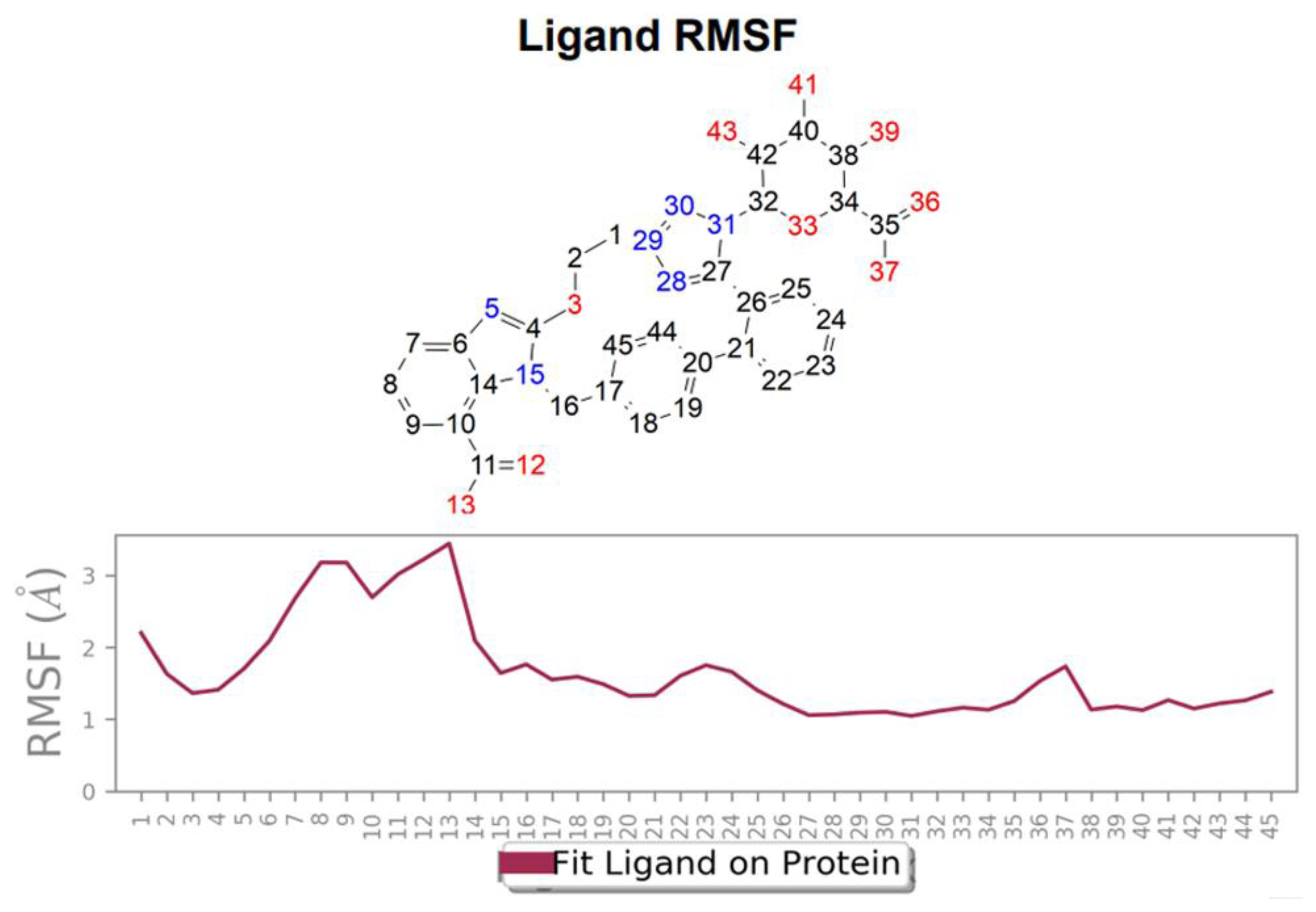

Supplement: Figure S14 — RMSF values of ligand coded 3665 during 1000-ns simulation. [file tjc-48-02-402s14.tif]

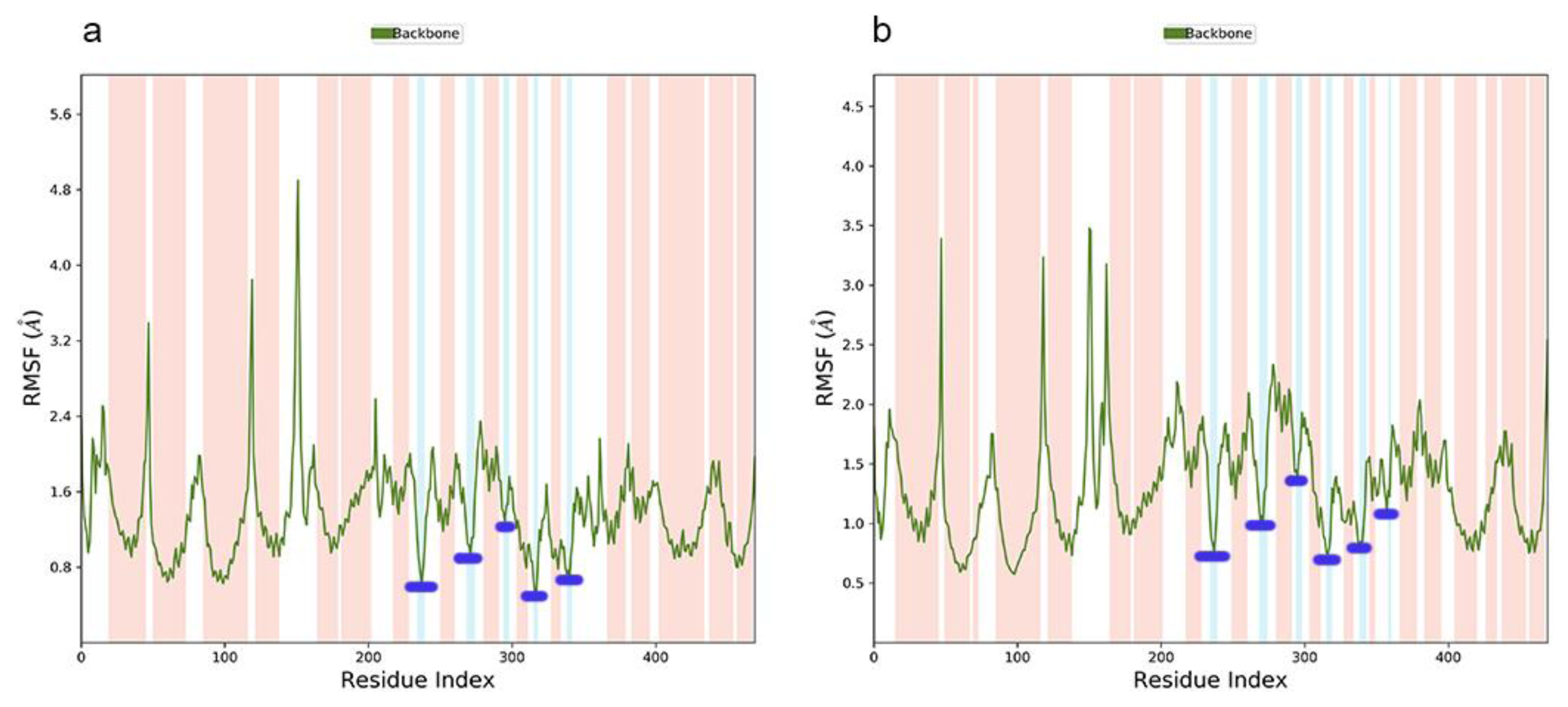

Supplement: Figure S15 — (a) RMSF values of a 3665-GnRH1R complex with seconder structure (light red), strands (light blue), and relationship of strands and protein structures dark blue line; (b) RMSF values of elagolix-GnRH1R complex with seconder structure (light red), strands (light blue), and relationship of strands and protein structures dark blue line. [file tjc-48-02-402s15.tif]

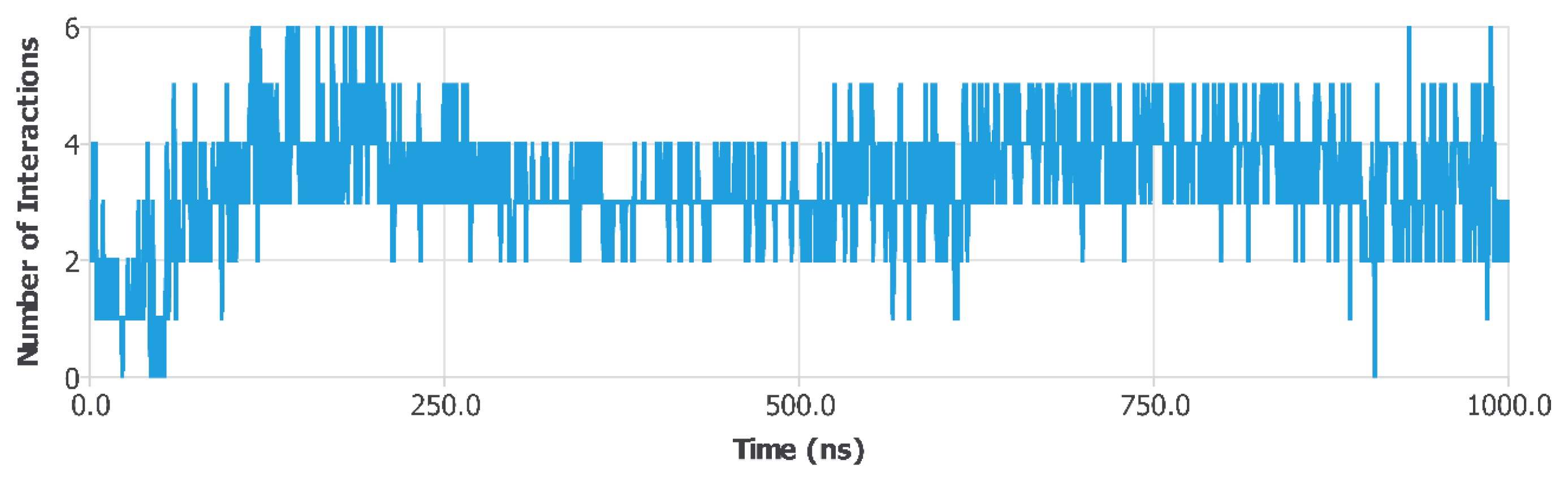

Supplement: Figure S16 — Variation of the number of hydrogen bonds of the ligand coded 3655 during the 1000-ns simulation. [file tjc-48-02-402s16.tif]

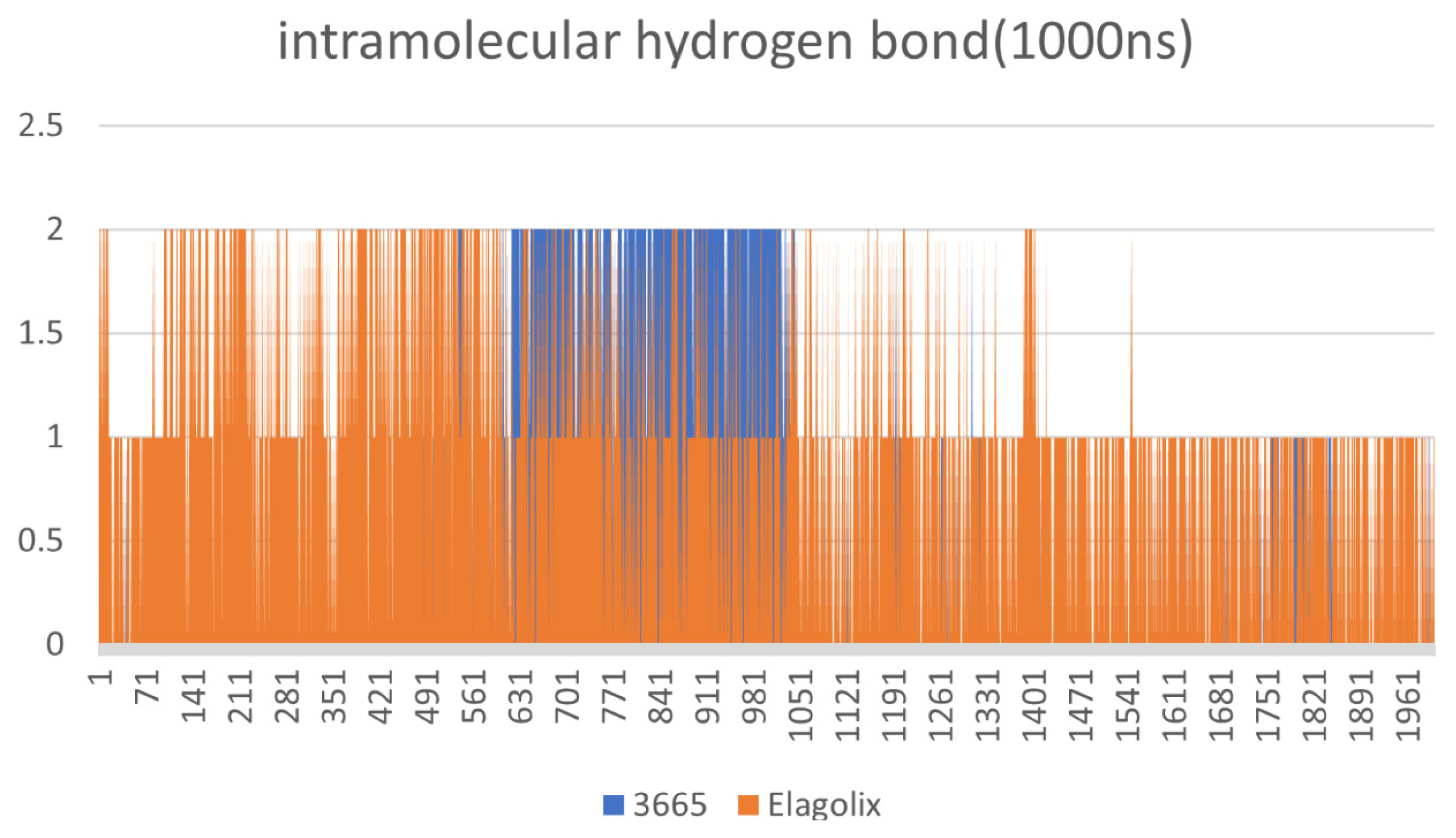

Supplement: Figure S17 — Variation of the number of intramolecular hydrogen bonds of elagolix and ligand coded 3665 with time. [file tjc-48-02-402s17.tif]
